# S‐Propargyl‐Cysteine Attenuates Stroke Heterogeneity via Promoting Protective Autophagy Across Multiple Neural Cell Types: Insights From Single‐Cell Sequencing

**DOI:** 10.1111/cns.70399

**Published:** 2025-07-24

**Authors:** Xiaoming Xin, Lei Miao, Lei Ci, Yun Wang, Zhiguo Zhang, Lingguo Meng, Jia Qi, Yicheng Mao, Yi‐Zhun Zhu

**Affiliations:** ^1^ School of Pharmacy, Shanghai University of Medicine and Health Sciences Shanghai China; ^2^ Department of Pediatric Surgery, Guangzhou institute of Pediatrics, Guangdong Provincial Key Laboratory of Researchin Structural Birth Defect Disease, Guangzhou Women and children's Medical Center, Guangzhou Medical University Guangzhou China; ^3^ School of Pharmaceutical Sciences, Departmentof Pharmacology, Shanghai Key Laboratory of Bioactive Small Molecules, Fudan University Shanghai China; ^4^ Shanghai Engineering Research Center for Model Organisms SRCMO/SMOC Shanghai China; ^5^ Shandong First Medical University Jinan China; ^6^ Department of Pharmacy, 960th Hospital of PLA Jinan China; ^7^ Department of Pharmacy, Xinhua Hospital Shanghai Jiaotong University School of Medicine Shanghai China; ^8^ School of Pharmacy Macau University of Science and Technology Macau China

**Keywords:** autophagy, cerebral ischemia–reperfusion, hydrogen sulfide, neuroprotection, S‐propargyl‐cysteine

## Abstract

**Introduction:**

Stroke, predominantly ischemic, is a leading cause of mortality and disability worldwide. Despite advances in intervention strategies, effective treatments to mitigate neurological injury post‐ischemic stroke remain limited. Hydrogen sulfide (H_2_S), a gas signaling molecule, has been implicated in neuroprotection, but its role in stroke is controversial. S‐propargyl‐cysteine (SPRC), an H_2_S donor, has shown great potential in protecting against neurological injuries, but its mechanisms in ischemic stroke are not fully understood. This study investigates the neuroprotective potential of SPRC and its mechanisms, focusing on the interplay between H_2_S and autophagy in modulating the cerebral microenvironment post‐stroke.

**Methods:**

We conducted a comprehensive single‐cell RNA sequencing analysis on ischemic brain tissue to elucidate the cellular heterogeneity and specific responses related to H_2_S synthesis and autophagy. We utilized the GEO repository dataset GSE174574, applying stringent filtering and batch effect correction using the Harmony R package. Cellular subpopulations were identified using established markers, and H_2_S and autophagy scores were calculated using the JASMINE package. We also measured serum H_2_S levels, evaluated the pharmacodynamics of SPRC in rats, and constructed a cerebral ischemia–reperfusion (I/R) injury model to assess the neuroprotective effects of SPRC. Additionally, we examined the role of SPRC in CBS and 3‐MST knockout mice to determine the dependency on these H_2_S synthetases.

**Results:**

Our findings revealed a dysregulation in the expression of H_2_S and autophagy‐related genes in central nervous system cells, particularly in neurons, following stroke. SPRC administration significantly improved neurological behavior, metabolic activity, reduced brain infarction size, and ameliorated ultrastructure changes in stroke‐affected rats. Interestingly, SPRC continued to provide neuroprotection even after the knockdown of CBS and 3‐MST, indicating a CBS/3‐MST‐independent mechanism. Furthermore, SPRC preserved the endogenous H_2_S level and strongly upregulated protective autophagy.

**Conclusion:**

This study is the first to reveal the neuroprotection of SPRC in cerebral I/R injury in a classical enzymatic CBS/3‐MST independent manner. The potential cellular and molecular mechanisms may rely on the promotion of SPRC to activated protective autophagy. Our results suggest that SPRC could be a promising therapeutic candidate for enhancing neuroprotection and modulating autophagy in ischemic stroke.

## Graphical abstract

Stroke is one of the major contributors to mortality and long‐term disability. Treatments aiming at regulating endogenous protective mechanisms to reduce neurological injury after ischemic stroke are still urgently needed. Herein, we investigated the neuroprotective potential of S‐propargyl‐cysteine (SPRC), a novel water‐soluble donor of H_2_S, and its possible mechanisms. Histologically, it was observed that the percentage of cells expressing hydrogen sulfide‐related genes in stroke‐affected brain tissues was lower compared to those in healthy brain samples. Autophagy, an emerging area of research in stroke studies, has been shown to modulate the cerebral microenvironment. Nevertheless, the relationship of hydrogen sulfide and autophagy levels remains elusive, which reason is partly due to the inherent heterogeneity in cellular populations present in stroke lesions. This investigation provides a thorough examination of the single‐cell transcriptomic profiles pertaining to hydrogen sulfide synthesis and autophagy across a spectrum of cellular phenotypes within ischemic brain tissue. Our findings reveal dysregulation in the expression of hydrogen sulfide and autophagy‐related genes, particularly in the central nervous system, such as microglial cells, astrocytes, glial cells, and neurons. Moreover, the significant increase in correlation between hydrogen sulfide and autophagy levels was greater in neurons of stroke compared to the control group. Experimental results showed that neurological behavior assays, metabolic activity, brain infarction size, and ultrastructure changes were significantly ameliorated by SPRC administration. After knockdown of cystathionine‐β‐synthase (CBS) and 3‐mercaptopyruvat sulfurtransferase (3‐MST), SPRC could still alleviate neurological injury. Meanwhile, SPRC could preserve the endogenous balance of H_2_S levels. Furthermore, protective autophagy was strongly upregulated by SPRC administration. Our results first revealed the neuroprotection of SPRC in cerebral I/R injury in a classical enzymatic CBS/3‐MST‐independent manner, and the potential cellular and molecular mechanisms may rely on the promotion of SPRC to the activated protective autophagy.

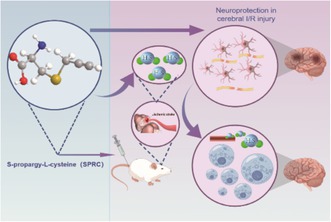

**Abbreviations**
3‐MA: 3‐Methyladenine
3‐MST: 3‐mercaptopyruvat sulfurtransferase
CBS: cystathionine‐β‐synthase
CSE: cystathionine‐γ‐lyase
DMEM: Dulbecco's modified Eagle medium
FBS: fetal bovine serum
Hcy: homocysteine
I/R: ischemia–reperfusion
LC 3‐II: LC 3‐phosphatidylethanolamine conjugate
LC3‐I: cytosolic light chain 3‐I
LDH: lactic dehydrogenase
MAP‐2: microtubule associated protein 2
MCAO: middle cerebral artery occlusion
OGD: oxygen glucose deprivation
ROS: reactive oxygen species
SPRC: S‐propargyl‐cysteine

## 1 Introduction

Stroke, a cerebrovascular disease, is largely caused by cerebrovascular thrombosis or a sudden burst of bleeding attributed to cerebral blood circulation disorders [1]. Stroke is the third leading cause of death and disability worldwide [2]. Due to the aging population and heavy burden of life, the incidence of stroke is rapidly increasing, especially in developing countries [3]. Even in developed countries, such as the United States, a person suffers from stroke every 40 s and one dies of stroke every 3 to 4 min on average [4]. Among different kinds of cerebrovascular diseases, 70%–80% is ischemic stroke. Current mainstay interventions for ischemic stroke focus on acute reperfusion therapies (e.g., intravenous thrombolysis and endovascular thrombectomy) to restore cerebral blood flow, supplemented by antiplatelet/anticoagulant therapies to prevent thrombus progression, alongside blood pressure management and neuroprotective agents (e.g., edaravone) to mitigate secondary injury. However, effective therapeutics are still insufficient and desiderata in clinical practice. Thus, it is worthwhile to explore effective drug candidates for better cerebral hypoxia tolerance and neuroprotection.

Hydrogen sulfide (H_2_S) has been recognized as the third gas signaling molecule in addition to carbon monoxide and nitric oxide [5, 6]. The endogenous H_2_S is produced mainly by three enzymes, namely, cystathionine‐γ‐lyase (CSE), cystathionine‐β‐synthase (CBS) and 3‐mercaptopyruvate sulfurtransferase (3‐MST). Although both CSE and CBS are mainly expressed in mammals, only CBS and 3‐MST dominantly distribute in the nervous system while CSE mostly allocates in the cardiovascular system [7, 8]. As a small gas molecule, H_2_S can penetrate blood brain barrier and cell membrane freely, which is a unique advantage for neuron disease intervention [9]. In the central nervous system, H_2_S not only functions as a neurotransmitter and a neuromodulator, but also involves learning, memory, and nociception [10]. Besides, H_2_S has been widely recognized as a neuroprotectant in several neurodegenerative and neurological damage diseases [7]. For example, the ameliorative role of H_2_S has been verified in memory and cognitive deficits induced by stress or trauma [11, 12, 13]. H_2_S also possesses alleviative potential in a mouse model of Alzheimer's Disease [14, 15]. However, the role of H_2_S in stroke remains controversial [16, 17]. Several studies have demonstrated that H_2_S plays a significant role in neuroprotection in stroke [18, 19, 20, 21]. However, its neurotoxicity has also been reported in other research [22, 23]. Under such a background, it is necessary for us to clarify the role of H_2_S in cerebrovascular disease, including ischemic stroke, in the defined research condition before further therapeutic exploration.

Although sodium hydrosulfide has been used as a routine exogenous H_2_S donor, its short half‐life, fast elimination, and inaccurate metering have directly limited its clinical translation [24]. Therefore, S‐propargyl‐cysteine (SPRC), a water‐soluble compound that endogenously produces H_2_S, has been developed as a promising drug candidate for H_2_S‐mediated treatment [25]. From previous studies, SPRC has been shown to alleviate learning and memory dysfunction induced by lipopolysaccharide in rats [26, 27]. Additionally, SPRC has been shown to be protective against myocardial ischemia and hypoxia with anti‐inflammatory and anti‐oxidative mechanisms [28, 29]. However, its pharmacological role and potential mechanisms in ischemic stroke remain obscure.

Autophagy refers to a tightly controlled process that includes the decomposition of the cell's own structure through lysosomal mechanisms. The main characteristic of autophagy is the formation of autophagosomes, and the maturation of autophagosomes is marked by the transformation of cytosolic light chain 3‐I(LC3‐I) to LC3‐phosphatidylethanolamine conjugate (LC3‐II) [30]. Physiologically, autophagy functions as a routine process in cell growth, development, and homeostasis, which maintains the cell production in a regulatory and balanced cycle status [31]. Autophagy also participates in and affects the pathophysiological processes of neurological diseases such as stroke, neurodegenerative diseases, and brain trauma [32, 33]. In ischemic stroke, it is undisputed that autophagy is activated [34, 35, 36, 37]. However, it is still controversial concerning the role of this activated autophagy: whether it is a friend or a foe [30]. Therefore, it is important to cautiously distinguish whether the activated autophagy is neuroprotection or neurodeath in the detailed background.

**SCHEME 1:**
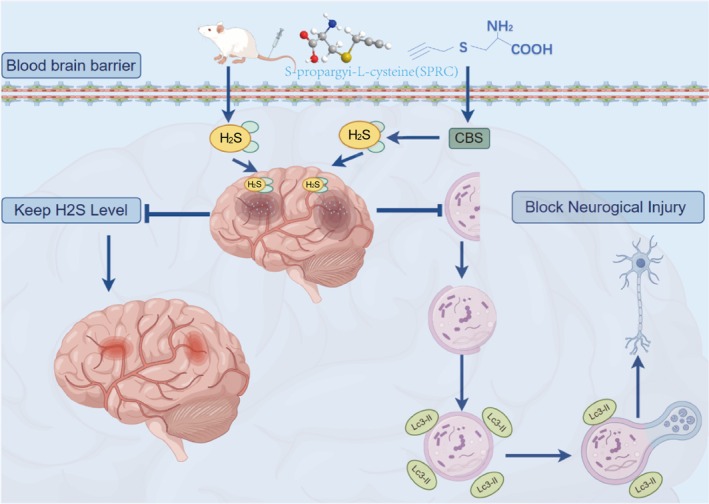
Schematic diagram of neuroprotection of S‐propargyl‐cysteine against cerebral ischemia–reperfusion injury.

Since H_2_S is involved in the regulation of cerebral pathophysiological homeostasis, in the current study, we performed integrated single‐cell analysis to reveal the relationship between H_2_S and autophagy across a spectrum of cellular phenotypes within ischemic brain tissue, and intended to investigate the pharmacological role of H_2_S donor SPRC in ischemic stroke in animals with cerebral ischemic‐reperfusion injury. Recent advancements in single‐cell RNA sequencing (scRNA‐seq) technology provide a powerful tool to dissect the cellular heterogeneity and specific responses of various brain cell types during ischemic conditions [38]. By analyzing the transcriptomic profiles of individual cells, researchers can uncover the distinct roles of H_2_S and autophagy in different cellular contexts, paving the way for targeted therapeutic strategies. This high‐resolution approach will allow us to dissect the heterogeneity of cellular responses to ischemic injury and provide insights into the underlying mechanisms driving neuroprotection and recovery. Furthermore, considering cellular autophagy has been revealed as an important pathological mechanism of ischemic stroke, the underlying mechanisms of action ascribed to SPRC have also been addressed with a focus on autophagy (Scheme 1).

## 2 Methods and Materials

### 2.1 scRNA‐Seq Data Preprocessing and Identification of Cellular Subpopulations

We accessed the single‐cell dataset labeled GSE174574 from the GEO repository (https://www.ncbi.nlm.nih.gov/). Initially, we meticulously cleaned the dataset, excluding cells that exhibited gene counts below 200, above 5000, or with mitochondrial genes exceeding 25%. This stringent filtering process culminated in a refined dataset comprising 58,505 cells for subsequent analysis. To mitigate any batch effects that might skew the subsequent analysis due to sample origin, we deployed the “Harmony” R package (version 0.1.0) for batch effect correction [39]. Post the correction, we conducted a t‐SNE (t‐distributed stochastic neighbor embedding) analysis to delineate the cellular clusters. This was achieved by applying the FindNeighbors and FindClusters algorithms with a resolution parameter set to 0.1. For the identification of these clusters, we relied on established markers that have been documented in the existing scientific literature [40]. Specifically, Astrocyte was represented by *Aldoc*, *Gfap*, *Aqp4*, *Slc1a3*; Endothelial cell expressed *Tie1, Tek, Vwf, Cldn5*; Glial cell expressed *Plp1, Sox10, Npy, Gja1*; Microglial cell expressed *Arpp21, Clec7a, Cx3cr1, Iba1, Ly6C, P2ry12, P2ry13, R3hdm1, Tmem119, Alf1, C1qa, Csf1r;* Neutrophils expressed *Cd44, Ccrl2, S100a9*; Myeloid cell expressed *Lyz2, Cd11b, Cx3cr1, Csf1r*; neuron expressed *Gad1, Gad2, Slc32a1, Slc17a6, Sp8, Tubb5*; NK cell expressed *Ncr1, Klrb1c, Klre1, Xcl1, Bcl2*; Pericytes expressed *Vtn, Rgs5, Kcnj8, Pdgfrb*.

### 2.2 Calculation of Cell Hydrogen Sulfide and Autophagy Score

We employed the ReactomeGSA tool from the R programming environment to assess the functional enrichment for each distinct cell type and investigate the biological mechanisms that underpin the functions of each MCAO scRNA‐Seq cell category. Additionally, the CytoTRACE software, specifically version 0.3.3, aided in forecasting the sequence of cell differentiation stages and offers valuable perspectives on cellular stemness [41]. To assess the Hydrogen sulfide (Systematic name: MM9615 and MM13529) and Positive regulation of autophagy (Systematic name: MM5430) enrichment score for each cell, we collected the genes from the GSEA database and calculated single‐cell scores by jointly assessing signature mean and inferring enrichment (JASMINE) from the JASMINE package [42], incorporating 16 selected H_2_S‐associated and 161 positive regulation of autophagy‐associated genes (Table S1). Finally, matrix bubble diagram and scatter diagram of the above two enrichment scores were performed through Hiplot (https://hiplot.com.cn/home/index.html).

### 2.3 Human Sample Collection

All human samples involved in the study were obtained according to the Eighty eighth Hospital of the People's Liberation Army ethics requirements, which was approved by the Medical Ethics Committee (No. 2016–010202). Blood samples from acute cerebral infarction patients were collected from the department of neurology and normal human samples were collected from department of physical examination center. Informed consent was obtained from all participants. For patients inclusion criteria, please refer to the “Eligibility Criteria for Participants” (S3).

### 2.4 H_2_S Measurement

The serum was collected 48 h after the cerebral ischemia operation. The serum H_2_S levels were measured according to a previous study [43]. Briefly, 30 μL serum was mixed with 10 μL Tris–HCL buffer (200 mM, pH 8.5) and 70 μL MBB solution (3.5 mM dissolved in acetonitrile). After shaking for 1 h in the dark, 10 μL 20% formic acid (v/v) was added and vortexed for 10 s. The supernatant was collected after 14,000 rpm for 10 min and stored at −70°C. Samples were measured by gas chromatography AB 4000Q TRAP tandem mass spectrometry detection [43]. Concentrations of H_2_S were determined in accordance with the standard curve.

### 2.5 SPRC Pharmacodynamics Study

All animal experimental protocols were approved by the institutional ethical committee according to internationally accepted ethical standards. The animals were supplied by the Laboratory Animal Centre, Fudan University, Shanghai, China. Male Sprague–Dawley (SD) rats (6–8 weeks), weighing between 280 and 320 g, were intravenously injected with SPRC (dissolved with Phosphate Buffered Saline) at three different dosage levels (6.25 mg/kg, 12.5 mg/kg and 25 mg/kg). The first tail vein injection was performed within 3 min after the surgery, followed by a repeat injection after 90 min. Blood samples were collected at 5, 15, 30, 60, 95, 105, 120, 180, and 240 min after injections. Serum was separated for H_2_S measurement as described above.

### 2.6 Cerebral Ischemia Reperfusion Model for Rats and Mice

Healthy 6–8 weeks male SD rats were housed under diurnal lighting and fed either food or water. Transgenic mice were obtained using C57BL/6J mice. The Longa ligation method was used to clog the left cerebral middle artery of rats or mice to develop the cerebral ischemia–reperfusion (I/R) injury [44]. After 1.5 h of ischemia, the suture was removed, the artery was ligated, and the muscle and skin were sutured. Animals were placed in a warm and comfortable environment until awake. All experimental operations were carried out under the premise of alleviating the suffering of animals. The rats or mice in sham groups only received anesthesia and artery separation without ligation.

The middle cerebral artery occlusion (MCAO) model was monitored and confirmed by Doppler flow detection (moor LDI2‐HIR, Moor Instruments, Britain) with a 2‐D laser speckle flow imaging technique. When the line blot was plugged into the beginning of the middle cerebral artery, the blood flow values suddenly dropped to 20%–30% of the original baseline (Figure S1A,B). It remained at such low blood flow during the whole process of cerebral I/R injury and gradually recovered almost close to the baseline until the bolt was slowly pulled out, which indicated that MCAO was successfully built up.

### 2.7 Construction of CBS ^+/−^ + 3‐MST‐Ckd Mice

Cystathionine‐β‐synthase (CBS) knockout mice in a C57BL/6J background were obtained from Shanghai Research Center for Model Organisms and backcrossed to a C57BL/6J background. Wild‐type control (CBS^+/+^) mice from the same litter were used at 8 and 10 weeks of age. All animals' genotyping was performed using primers and protocols provided by the supplier. The CBS‐KO mice were generated by standard embryonic stem cell (ESC) based gene targeting techniques and verified by PCR and southern blot. Briefly, the targeting vectors contained homologous recombination CBS gene fragments (Figure S2A) were transferred into ESC, and then the ESC with homologous vectors were transplanted into the blastosphere to breed chimeric mice. CBS knockout mice were genotyped from tails using PCR (forward: 5′‐CCCTGGCATAGTCTCACAA‐3′; reverse: 5′‐ACTAGAGCTTGCGGAACCC‐3′) and southern blot. The identification bands indicated that non‐transgenic mice (NGT, namely wild type) only presented at 355 bp, while the homozygous (CBS^−/−^) mice presented at a 203 bp band. The heterozygous CBS^+/−^ mice presented at both 355 bp and 203 bp (Figure S2B). CBS^−/−^ mice all died at the embryo or within 24 h after birth, which was consistent with the previous research [45]. CBS^+/−^ mice were smaller than NGT mice in the first 4 weeks in size, but there was no difference after adulthood. No other obvious difference was observed in appearance and movement between the two groups. The brain tissues were stained with Indian ink, and no difference was found in cerebral arteries anatomy in NGT and CBS^+/−^ mice (Figure S2C).

The 3‐MST shRNA lentivirus, purchased from OBiO Technology (Shanghai) Co. Ltd., was injected into the brains of CBS^+/−^ mice using a stereotaxic device as previously described [46]. We injected lentivirus into the cerebral cortex of mice with 1 μL lentivirus injected into each half of the brain at the coordinates (BLA, AP: −2 ± 0.5 mm; ML, ±1 mm; DV, −0.6 mm), and then we collected the cerebral cortex between the anterior and posterior fontanelles 2 weeks later for detection. The mice could be used for subsequent experiments.

The physiological characters of CBS^+/−^‐3MST‐cerebral knockdown (CBS^+/−^ + 3‐MST‐ckd) were measured and compared to the NTG and CBS^+/−^ mice, and no significant difference was found in these mice (Figure S3D,E and Supporting Table S1).

### 2.8 Animal Experiment Groups for SPRC Therapy Study

Male SD rats were divided randomly into the following groups: sham‐operated group, cerebral I/R injury model group, SPRC groups (6.25, 12.5, 25 mg/kg, *n* = 47 per dosage group), and positive control edaravone group (3 mg/kg, *n* = 20). The administration method of SPRC has been described in Section 2.3. Edaravone was administered as a single intravenous injection within 3 min after modeling at a dose of 3 mg/kg.

Male mice were divided randomly into the 8 following groups: NTG + sham‐operated group (*n* = 6), NTG + CIRI model group (*n* = 7), CBS^+/−^ + I/R group (*n* = 8), CBS^+/−^ + 3‐MST‐ckd + I/R group (*n* = 6), CBS^+/−^ + 3‐MST‐ckd + I/*R +* SPRC low dosage group (20 mg/kg, *n* = 6), CBS^+/−^ + 3‐MST‐ckd + I/*R* + SPRC middle dosage group (40 mg/kg, *n* = 6), CBS^+/−^ + 3‐MST‐ckd + I/*R* + SPRC high dosage group (80 mg/kg, *n* = 7) and CBS^+/−^ + 3‐MST‐ckd + I/*R* + L‐Cys (30 mg/kg, *n* = 6) as a positive control. The scale of surgery and dosing refers to Figure S2C.

### 2.9 Brain Infarction Size Measurement

48 h after reperfusion, animals were sacrificed and the whole brain was collected and quickly frozen at −80°C for 5 min. The brain tissues were coronally cut into a set of continuous 2 mm slices. The infarct area was observed by using 0.2% TTC staining at 37°C for 30 min, followed by fixing in 4% paraformaldehyde overnight. Normal brain tissues were stained fresh red, while the infarcted area was pale. The Image J software (NIH, Boston, MA) was used to analyze the infarct volume. Infarct volume (%) = 100% × infarct area/total area of the brain slice.

### 2.10 Behavioral Evaluation

The neuronal function impairment was evaluated based on a neurological deficit grading system with a scale of 0 to 7 as previously described [47] at 48 h after ischemic stroke.

Spontaneous motor activity was measured using a rota‐rod treadmill for rats, under 10 speeds from 4 to 40 rpm for 5 min. The interval from the timepoint when the animal climbed the rod to that when it fell down was recorded as the performance time [48]. The animals were trained five times per day until 2 days before cerebral IR injury in order to obtain stable baseline values. The mean duration for 5 times' training was recorded.

Cat walk evaluation was carried out as the following steps. Rats were trained to walk across a glass aisle daily for 1 week before surgery. Training was resumed 24 h after surgery, and data were evaluated 48 h after surgery.

### 2.11 PET/CT Scanning

Animals were anesthetized by i.p. 60 mg/kg pentobarbital sodium 48 h after surgery and fixed on a plate. Intravenously injected with F‐18‐FDG, the animal was sent into PET‐CT for 45 min for imaging. CT scanned for 10 min: 80 kV, 500 μA, exposure time 1100 ms, 600 s/round, Fov (mm): 768 × 768 × 512. PET scanned for 20 min: Fov (mm): 128 × 128 × 159. Using the 3D acquisition method and all the cross‐sectional images were selected. Images were analyzed by utilizing Inveon Acquisition Workplace software.

### 2.12 Brain Water Content

48 h after surgery, rats or mice were anesthetized and their brains on the ischemic side were separated into hemispheres. The wet weight was immediately measured and recorded. After drying at 60°C for 48 h, the dry weight of the same samples was quantified by an electronic analytical balance. The formula for the brain water content calculation was as follows: (wet weight‐dry weight) / wet weight × 100%.

### 2.13 Primary Neuronal Culture

Cell plates were precoated with 0.1 mg/mL poly‐lysine in advance for 12–24 h and blowdried sterilely. Pregnant rats (16–18 days) were sacrificed, and fetal rats were removed to high glucose Dulbecco's modified Eagle medium (DMEM, Hyclone, USA) without fetal bovine serum (FBS). The cerebral cortex of fetal rats was separated in buffer and washed with hank's buffer 3 times. Hank's buffer was later replaced by trypsin. Tissues were cut with a scissors and incubated at 37°C for 30 min after being transferred into high glucose DMEM medium added with 10% FBS, and a pipette was used to gently blow into a single neuronal cell. After centrifuging at 800 rpm for 8 min, the precipitated cells were counted and plated in the prepared cell culture plates. The primary neurons were identified by microtubule‐associated protein 2 (MAP‐2) staining and the purity was > 95% (Figure S4).

### 2.14 MTT Assay

Neurons were planted and incubated in a 96‐well plate after 7 days' primary culture, and the supernatant was replaced with DMEM without glucose or FBS to develop an oxygen glucose deprivation (OGD) model. 3‐Methyladenine (3‐MA), the specific inhibitor of autophagic/lysosomal protein, was used to suppress autophagy. The OGD condition and the corresponding treatment groups were kept at the settled different time points and changed to normal medium for additional 2 h incubation. In each well, 20 μL MTT (final concentration is 0.5 mg/mL) were added and incubated for 4 h at 37°C with 5% CO_2_. After removal of supernatant, samples were washed with D‐Hank's solution once followed by 100 μL DMSO and 10 min slight shaking. The absorbance was detected by a microplate reader at 490 nm (Infinite 1000; Tecan, Switzerland).

### 2.15 Lactate Dehydrogenase (LDH) Assay

Primary neurons were planted at 10^4^ cells/ml in a 96‐well plate on the seventh day. After overnight incubation, the medium was replaced with DMEM with 0.5% FBS. Cells were incubated in low serum medium and then exposed to the corresponding reagents for 30 min. The plates were centrifuged at 400 g for 5 min and the supernatants were transferred into a new 96‐well plate for detection following the protocol from the manufacturer (LDH cytotoxicity assay kit, Beyotime Biotechnology, Shanghai, China) at 490 nm, and calculation was performed in accordance with the instructions.

### 2.16 Western Blot Analysis

Tissue lysates were prepared using RIPA lysis buffer (Beyotime Biotechnology, Shanghai, China) containing protease and phosphatase inhibitors. Immunoblot was used to analyze proteins with a standard procedure. Briefly, electrophoresis was used to separate proteins in a sodium dodecyl sulfate polyacrylamide gel and then transferred to a nitrocellulose membrane. The membrane was incubated with different primary antibodies at 4°C overnight after blocking. After incubation with peroxidase‐conjugated secondary antibody (HRP fluorescent anti‐mouse or anti‐rabbit from Cell Signaling Technology) for 2 h at 37°C, the membrane was imaged using the Odyssey two‐color infrared laser imaging system (LI‐COR Biosciences, Lincoln, NE, USA). The relative intensity was calculated with ImageJ (NIH).

### 2.17 Immunofluorescent Staining

Sample was washed three times with PBS and incubated in 0.3% Triton‐X 100 solution for 15–30 min, and an additional three times washing with PBS was performed again. Non‐specific binding points were blocked by blotting buffer for 1 h, and then incubated with primary antibody at 4°C overnight. After washing with PBS for three times, the sample was incubated with 1:200 diluted fluorescent secondary antibody (Alexa Fluor 488 or 594) for 2 h before being stained with 1 μg/mL DAPI dye for 5 min. Cyto‐ID autophagy detection kit obtained from Enzo Life Sciences (Farmingdale, NY, USA) was used as described in the manufacturer's instruction. The slide was sealed after dehydration and was observed with a confocal microscope (Becton, Dicknson and Company, New Jersey, USA).

### 2.18 Transmission Electron Microscopy

The primary neurons were analyzed with a JEM 1230 transmission electron microscope (JEOL, USA Inc). Micrographs were taken at ×5,000 or ×20,000 magnification.

### 2.19 Statistical Analysis

Statistical analysis of the rat and mouse experimental data was conducted utilizing SPSS version 25 (IBM Corporation, USA), while GraphPad Prism version 9.0 (GraphPad Software Inc.) was employed for the generation of statistical charts. Data presentations were standardized to the format of mean values with their corresponding standard error of the mean (SEM). The Shapiro–Wilk test served as the method for assessing data normality. When data complied with the criteria for both variance homogeneity and normal distribution, the analysis was conducted by one‐way ANOVA with Tukey's HSD was implemented. Statistical significance was determined at a threshold of *p* < 0.05.

## 3 Results

### 3.1 Retrieving and Preprocessing of MCAO scRNA‐Seq Data

After preprocessing the data, we employed the harmony algorithm to integrate the samples and effectively remove any potential batch effects. Figure S1A illustrates the representation of the integrated dataset consisting of 3 MCAO and 3 control samples after implementing the harmony algorithm. Following the standard steps of Seurat, we successfully identified a total of 16 clusters, which were visualized using t‐SNE, as shown in Figure S1C,D. Then, we identified marker genes in the cell‐type populations (Figure S1B). We observed 9 cell clusters (Endothelial Cells: *Itm2a*, *Cldn5*, *Slco1a4*; Microglial cell: *Hexb*, *P2ry12*, *Selplg*; Myeloid cells: *Lyz2*, *Cd74*, *H2‐Ab1*; Astrocyte: *Aldoc*, *Clu*, *Mt3*; Pericytes: *Acta2*, *Tagln*, *Myl9*; Glial cell: *Plp1*, *Ptgds*, *Cldn11*; Neutrophils: *S100a8*, *S100a9*, *Retnlg*; NK cells: *Ccl5*, *Nkg7*, *Ms4a4b*; neuron: *Mgp*, *Igfbp6*, *Nov*).

### 3.2 Profiling of Cells in MCAO at the scRNA Transcript Level and Cell Stemness Analysis

**FIGURE 1:**
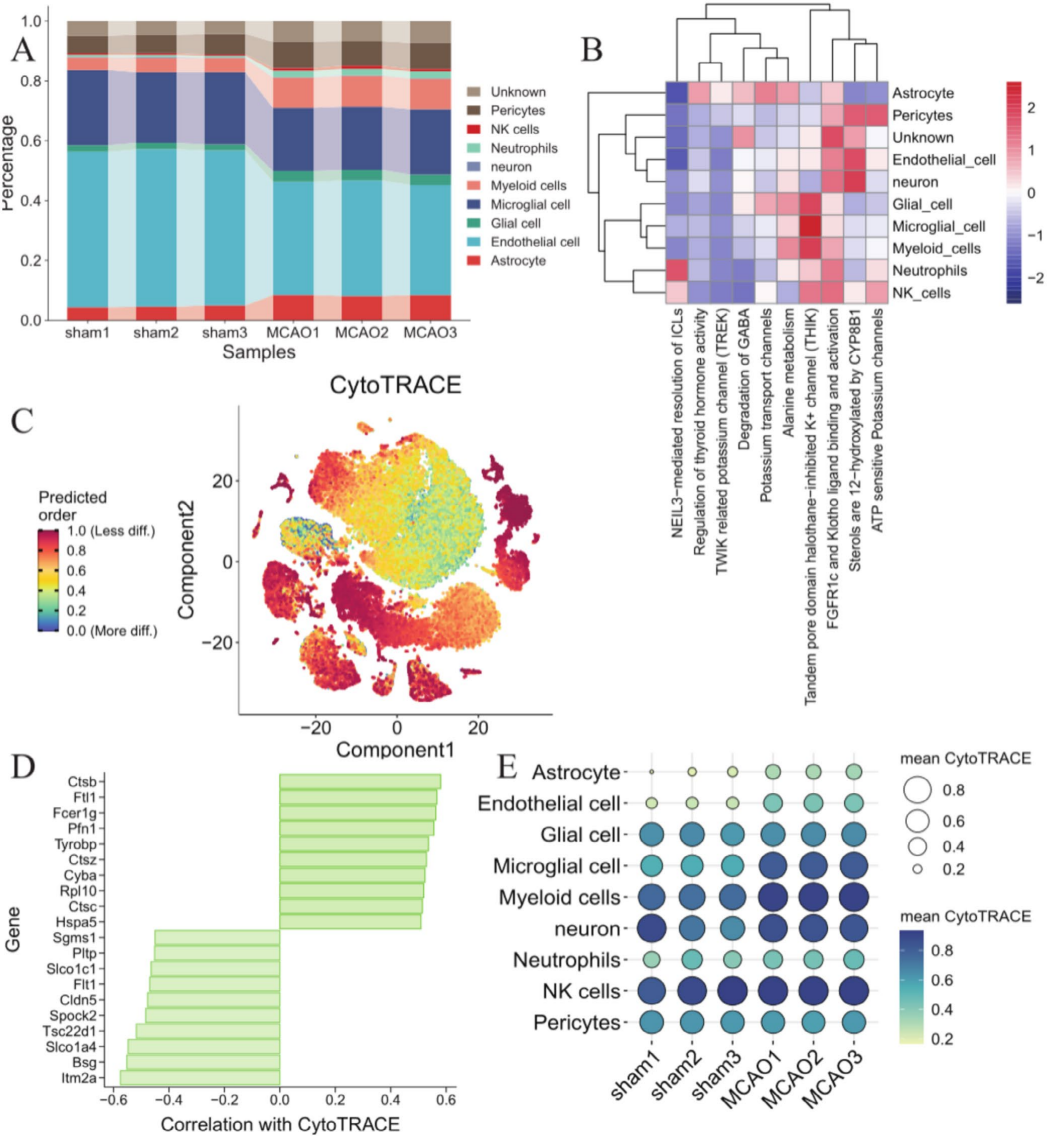
ScRNA‐seq data was used to annotate and analyze the distribution of cell proportions between MCAO and control samples. (A) The overall cell fraction between the two groups. (B) Pathway enrichment analysis of MCAO. (C) t‐SNE visualization is utilized to illustrate the level of stemness in each cell predicted by CytoTRACE. (D) Top 10 Genes related to CytoTRACE score. (E) Ballon plot showing the mean CytoTRACE score of each cell type in MCAO and control groups.

The distribution of cell types was compared between the MCAO and control samples. A significant difference was detected in various types, including astrocytes, glial cells, Endothelial Cells, and myeloid cells (Figure 1A). Figure 1B showed that MCAO progression was mainly associated with NEIL3‐mediated resolution of *ICLs*, Regulation of thyroid hormone activity, *TWIK‐related* potassium channel (*TREK*), Degradation of GABA, Potassium transport channels, Alanine metabolism, Tandem pore domain halothane‐inhibited K^+^ channel (*THIK*), *FGFR1c* and *Klotho* ligand binding and activation, and sterols are 12‐hydroxylated by *CYP8B1*, ATP‐sensitive Potassium channels.

By comparing the differences in CytoTRACE scores of cells between MCAO and control groups, we observed that Astrocyte, Endothelial cell, Microglial cell, and neuron showed a lower differentiated state and possessed a higher degree of stemness in MCAO than in control groups (Figure 1C,E). In addition, we identified the top10 genes with the highest correlation out of CytoTRACE scores, including *Ctsb*, *Ftl1*, *Fcer1g*, *Pfn1*, *Tyrobp*, *Itm2a*, *Bsg*, *Slco1a4*, *Tsc22d1*, *Spock2* (Figure 1D).

### 3.3 Calculation of Cell H_2_S and Autophagy Score

Since studies indicated that H_2_S and autophagy played a significant role in neuroprotection in stroke, we further explored whether H_2_S and autophagy define the different cell fate in MCAO. Figure S1 showed that Neutrophils, Myeloid cells, Microglial cells, Astrocytes, and Glial cells displayed a higher hydrogen sulfide level than other cell types. In Neutrophils, Myeloid cells, and Microglial cells, the H_2_S level of the MCAO group was significantly lower than that of the control group.

**FIGURE 2:**
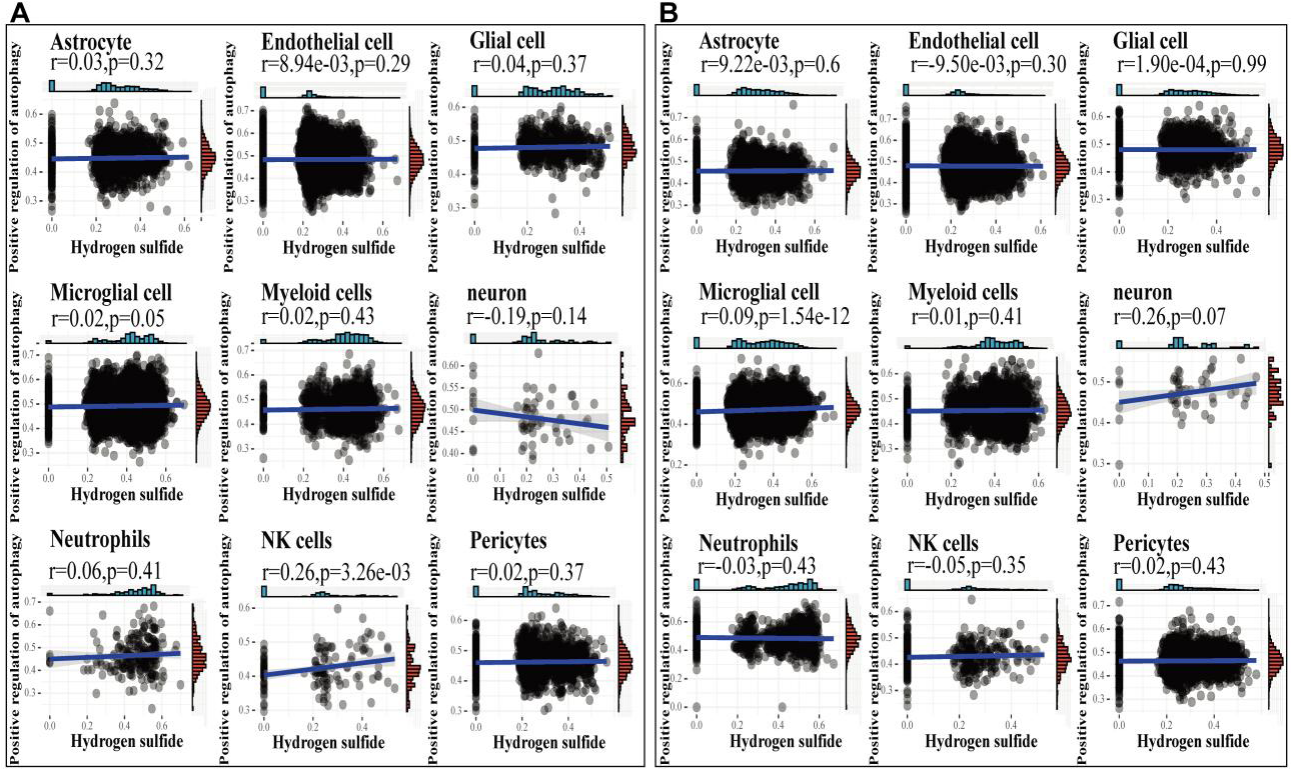
Correlation analysis. (A) Correlation analysis between Hydrogen sulfide and Positive regulation of autophagy scores in MCAO group. (B) Correlation analysis between Hydrogen sulfide and Positive regulation of autophagy scores in sham group.

Furthermore, through the correlation analysis between H_2_S and positive regulation of autophagy scores, we found the positive correlation in NK cells of sham (*r* = 0.26, *p* = 3.26e‐03, Figure 2A) and Microglial cell of MCAO (*r* = 0.09, *p* = 1.54e‐12, Figure 2B). Moreover, the significant increase in correlation was greater in neurons of MCAO (*r* = 0.26, *p* = 0.07) compared to the control (r = −0.19, *p* = 0.14) (Figure 2B), suggesting the crosstalk of H_2_S and positive regulation of autophagy plays a key role in neurons.

### 3.4 H_2_S Involves in the Pathophysiology of Ischemic Stroke

**FIGURE 3:**
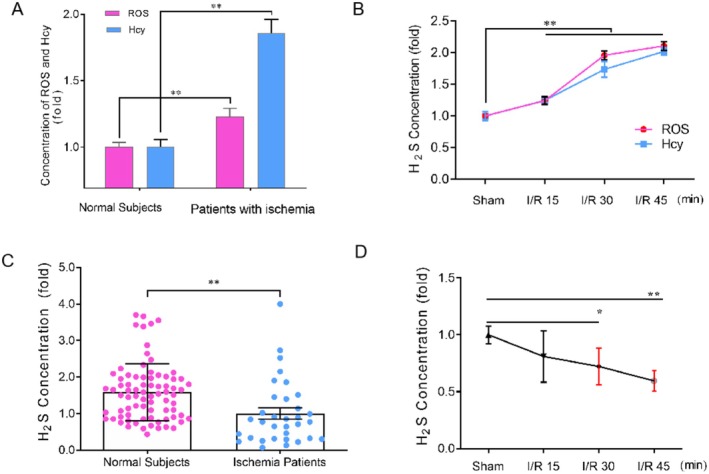
Changes of ROS, Hcy, and H_2_S concentrations in people and rats. Serum ROS, Hcy, and H_2_S were assayed. (A) Serum ROS and Hcy levels in normal and stroke people. (B) Serum ROS and Hcy levels in rats before and after cerebral ischemia/reperfusion (I/R) injury. (C) H_2_S concentration in normal subjects and stroke patients. (D) H_2_S concentration in rat serum at different timepoints after cerebral I/R injury. Data are mean ± sem. Comparison between two groups in (A) and (C) are analyzed using *t*‐test; in each group, *n* ≥ 10; While variance among multiple groups in (B) and (D) is assessed by One‐way ANOVA with Tukey's HSD, *n* = 6 per group. Compared with the normal/sham group, *p < 0.05, and **p < 0.01.

Homocysteine (Hcy) has been recognized as a risk factor for stroke [49], and increased reactive oxygen species (ROS) is both a foreshadow and a pathological marker of stroke [49]. Although H_2_S has been demonstrated as a pivotal signaling molecule in the central nervous system, no study has shown the age‐dependent changes of H_2_S in vivo so far. Due to the lipid solubility of H_2_S, the serum level of H_2_S can sufficiently reflect its concentrations in the brain [50]. Herein, in order to verify the above bioinformatics result and whether H_2_S is a potential risk factor like Hcy and ROS or a pathophysiological modulator in ischemic stroke, we measured the content of serum H_2_S, Hcy, and ROS in normal subjects and rats with different ages. As shown in Tables 1 and 2, H_2_S, Hcy, and ROS in normal subjects and rats exhibited an appreciable age‐dependent up‐regulation, seemingly implying H_2_S might be a risk factor like Hcy and ROS. However, in contrast to the age‐dependent up‐regulation of Hcy and ROS in ischemic patients and cerebral I/R injury rats (Figure 3A,B), the H_2_S level declined dramatically in acute ischemic stroke patients and cerebral I/R injury rats (Figure 3C,D, *p < 0.05, **p < 0.01), indicating that H_2_S may act as a pathophysiological modulator rather than a risk factor like Hcy and ROS in ischemic stroke.

**TABLE 1:** Changes of ROS, Hcy, and H_2_S in human serum from different ages.

Age (years)	10 ± 2	20 ± 2	30 ± 2	40 ± 2	50 ± 2	60 ± 2	70 ± 2	80 ± 2	*p*‐value (vs. 10 ± 2)
ROS	1.00 ± 0.25	1.50 ± 0.20	1.62 ± 0.31	1.64 ± 0.26	1.88 ± 0.19	1.73 ± 0.23	2.09 ± 0.16	2.18 ± 0.21	< 0.001
Hcy	1.00 ± 0.19	1.02 ± 0.18	1.14 ± 0.21	1.33 ± 0.10	1.50 ± 0.12	1.53 ± 0.11	1.66 ± 0.20	1.66 ± 0.16	< 0.001
H_2_S	1.00 ± 0.27	0.98 ± 0.19	0.92 ± 0.11	1.55 ± 0.55	1.59 ± 0.40	1.37 ± 0.54	1.61 ± 0.28	1.66 ± 0.44	< 0.001

*Note:* In each group, *n*=8‐19, data are presented as mean ± sem. Compared with the group 10 ± 2 (years), *p*‐values of groups 20 ± 2 to 80 ± 2 are all less than 0.001.

**TABLE 2:** Changes of ROS, Hcy, and H_2_S in rat serum from different ages.

Age (weeks)	12	24	36	48	60	72	84	96	*p*‐value (vs. 12)
ROS	1.00 ± 0.38	1.02 ± 0.12	1.57 ± 0.13	1.37 ± 0.14	1.85 ± 0.08	1.85 ± 0.10	2.04 ± 0.12	2.08 ± 0.14	< 0.001
Hcy	1.00 ± 0.13	1.18 ± 0.22	1.56 ± 0.35	1.24 ± 0.13	1.99 ± 0.24	1.73 ± 0.16	2.07 ± 0.32	2.29 ± 0.20	< 0.001
H_2_S	1.00 ± 0.08	1.10 ± 0.11	0.98 ± 0.02	1.25 ± 0.12	1.13 ± 0.16	1.33 ± 0.08	1.34 ± 0.11	1.38 ± 0.07	< 0.001

*Note:* In each group, *n* = 6, data are presented as mean ± sem. Compared with the group 12 (weeks), *p*‐values of groups 24 (weeks) to 96 (weeks) are all less than 0.001.

### 3.5 SPRC Blocks Deterioration of Cerebral Function After Rat Cerebral I/R Injury

**FIGURE 4:**
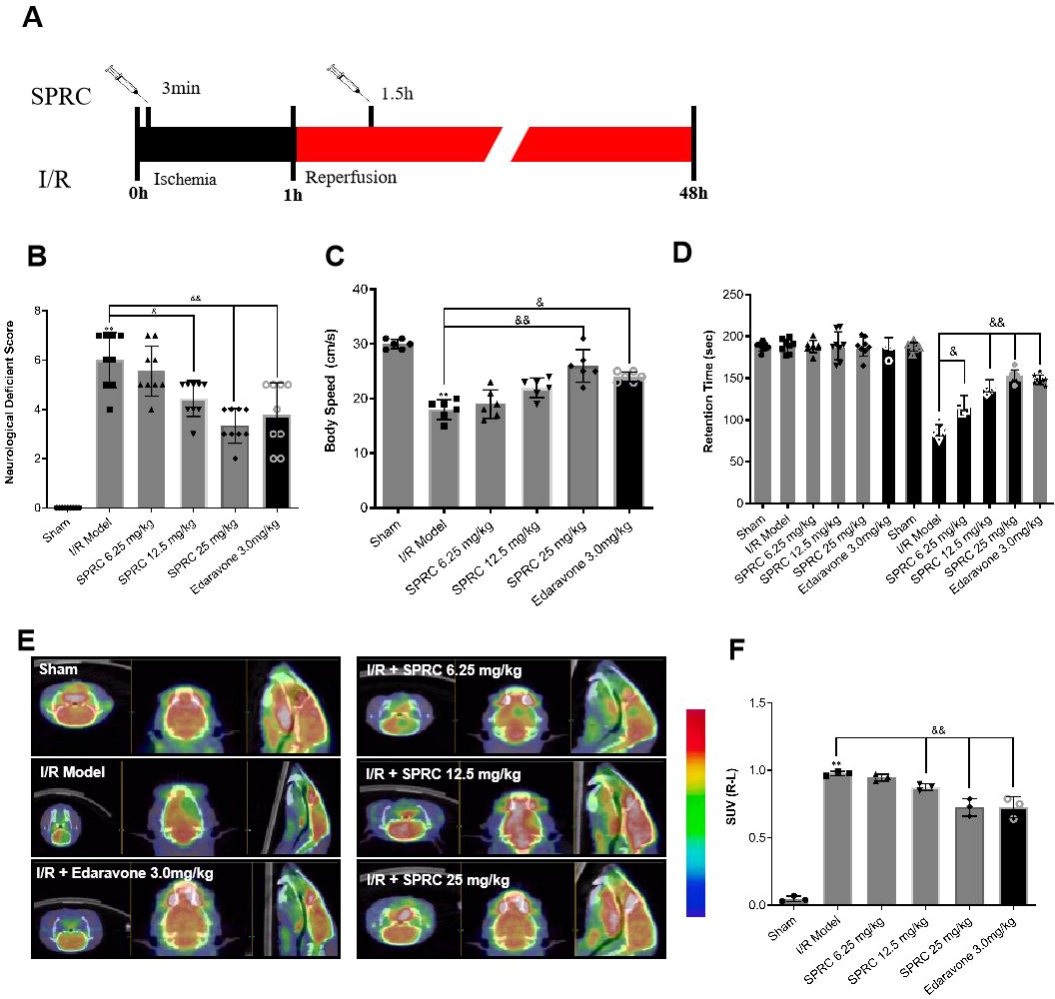
Administration of SPRC blocks deterioration of cerebral function after I/R injury. Rats suffered from middle cerebral artery occlusion and were treated with SPRC at a given time before functional parameter evaluation. (A) Time course of SPRC treatment to I/R rats. (B) Neuroethology score of rats in different groups. (C) Analytical data of cat walk evaluation. (D) Rotarod test in each experimental group. (E) Representative pictures of glucose metabolism in rat brains detected by micro‐PET. (F) Statistical data of micro‐PET assay in different groups. Data are presented as mean ± sem, one‐way ANOVA. Compared with the I/R group, ^&^ *p* < 0.05, ^&&^ *p* < 0.01.

As an endogenous H_2_S modulator, SPRC was widely studied in cardiovascular diseases including ischemic heart disease in rat models. But the therapeutic efficacy of SPRC on cerebral I/R injury has yet to be elucidated. In order to verify whether SPRC could protect against cerebral I/R injury, rats were treated with designed dosage SPRC at defined timepoints (Figure 4A). As shown in Figure 4B, compared to the sham group, rats in the I/R group gained an apparently high neurological deficient score, but SPRC at medium and high dosages could significantly reduce the ethological score like Edaravone treatment when compared with the I/R group (^&^ *p* < 0.05, ^&&^ *p* < 0.01). Similarly, another ethological indicator, body speed, was also accelerated by SPRC, especially at high dosage (Figure 4C, ^&&^ *p* < 0.01). Furthermore, retention time was largely reduced by SPRC and Edaravone treatment after training (Figure 4D, ^&^ *p* < 0.05, ^&&^ *p* < 0.01). In addition, the Micro PET results disclosed that SPRC, especially at medium and high dosages, significantly increased the standard uptake value (SUV) in the brain compared with the I/R model group, indicating that SPRC could promote the glucose metabolism (Figure 4E,F, ^&&^ *p* < 0.01). These data in living body demonstrated the protective potential of SPRC in cerebral I/R injury rats.

### 3.6 SPRC Ameliorates Neurological Injury Induced by Cerebral I/R Injury in Rats

**FIGURE 5:**
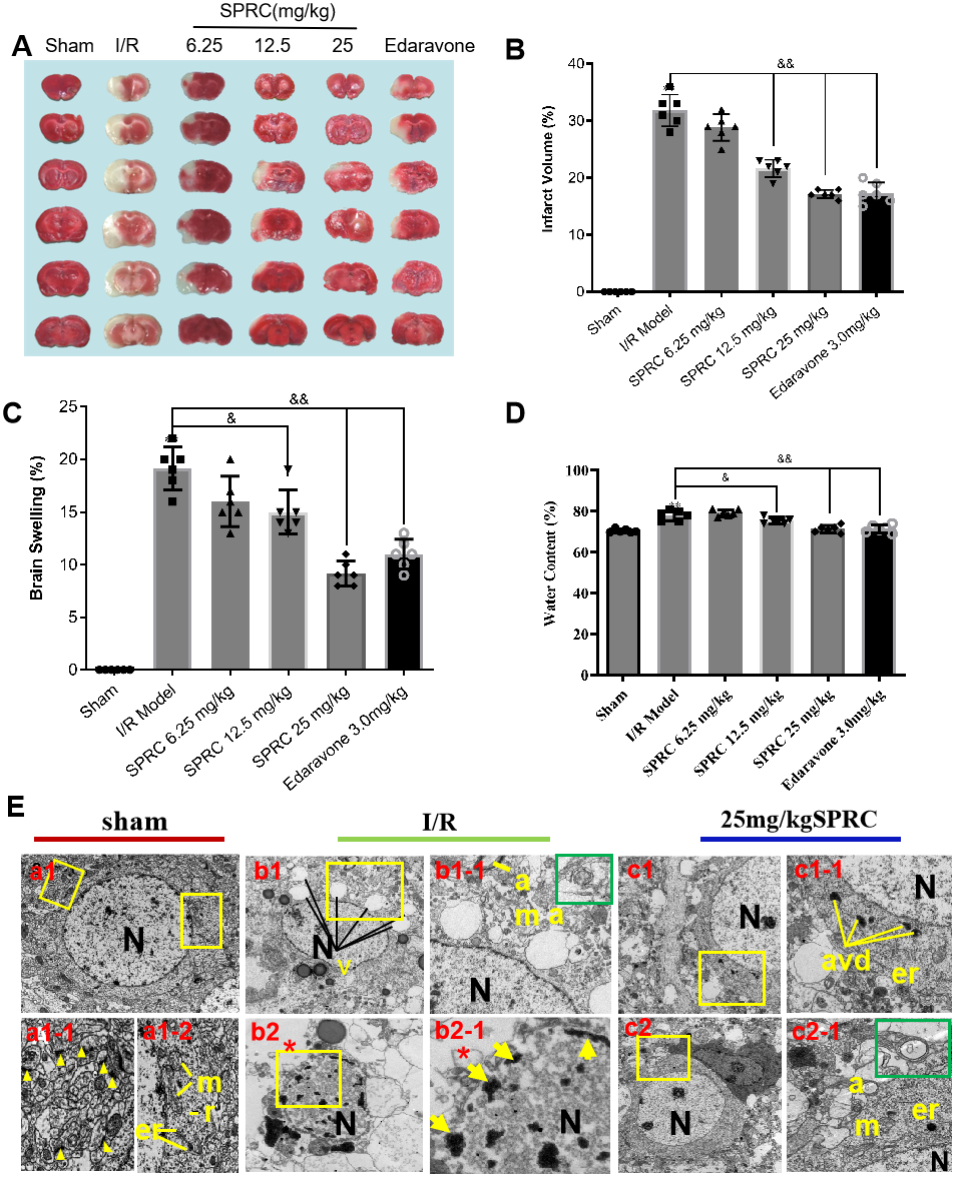
Administration of SPRC ameliorates neurological injury induced by I/R. Brain tissues were harvested after 48‐h I/R. (A) Representative graphs of TTC staining for cerebral tissues; Scale bar: 1 mm. (B) Analytical data for cerebral TTC staining. (C) Evaluation of rat brain swelling in each group. (D) Water content assay for different experimental groups. (E) Microscopic morphology changes detected by transmission electron microscopy; Scale bars: 1 μm (a1, b1, b1‐1, b2, c1, c1‐1, c2); 0.5 μm (a1‐1, a1‐2, b2‐1, c2‐1); *, autophagic electron‐dense substances; ▲, synapse; a, autophagosome; er, endoplasmic reticulum; m, mitochondria; N, nucleus; r, ribosome. Data are presented as mean ± sem, one‐way ANOVA. Compared with the I/R group, ^&^ *p* < 0.05, ^&&^ *p* < 0.01.

To further assess the neuroprotective potential of SPRC against ischemic stroke, we next evaluated other indicators at autopsy. Infarct volume was assayed by TTC staining. As presented in Figure 5A,B, when compared to the sham group, the infarct area and percentage of infarct volume in the I/R group were remarkably increased, while SPRC administration could significantly decrease the infarct volume (^&&^ *p* < 0.01), which was similar to the effect of positive control Edaravone (^&&^ *p* < 0.01). Detection of brain swelling and water content can indirectly reflect the degree of injury after I/R; in the I/R group, the percentage of brain swelling and water content were both largely increased compared to the sham group; however, after SPRC treatment, these two indicators were greatly reduced (Figure 5C,D, ^&^ *p* < 0.05, ^&&^ *p* < 0.01). We finally further evaluated the ultrastructure changes of brain tissues using transmission electron microscopy. As the graphs in Figure 5E show, in the sham group, the neuronal nucleus in the rat hippocampus was intact and regular in morphology with homogeneous nuclear chromatin, and the neurons were rich in synapses, mitochondria, endoplasmic reticulum, and ribosomes. However, after I/R injury, all of these ultrastructure manifestations SPRC administration could strongly salvage the ultrastructure deterioration. Summarily, these data further demonstrated the definite neuroprotection of SPRC against cerebral I/R injury.

### 3.7 Neuroprotection of SRPC Is Not Completely Dependent on Synthetase CBS and 3‐MST

**FIGURE 6:**
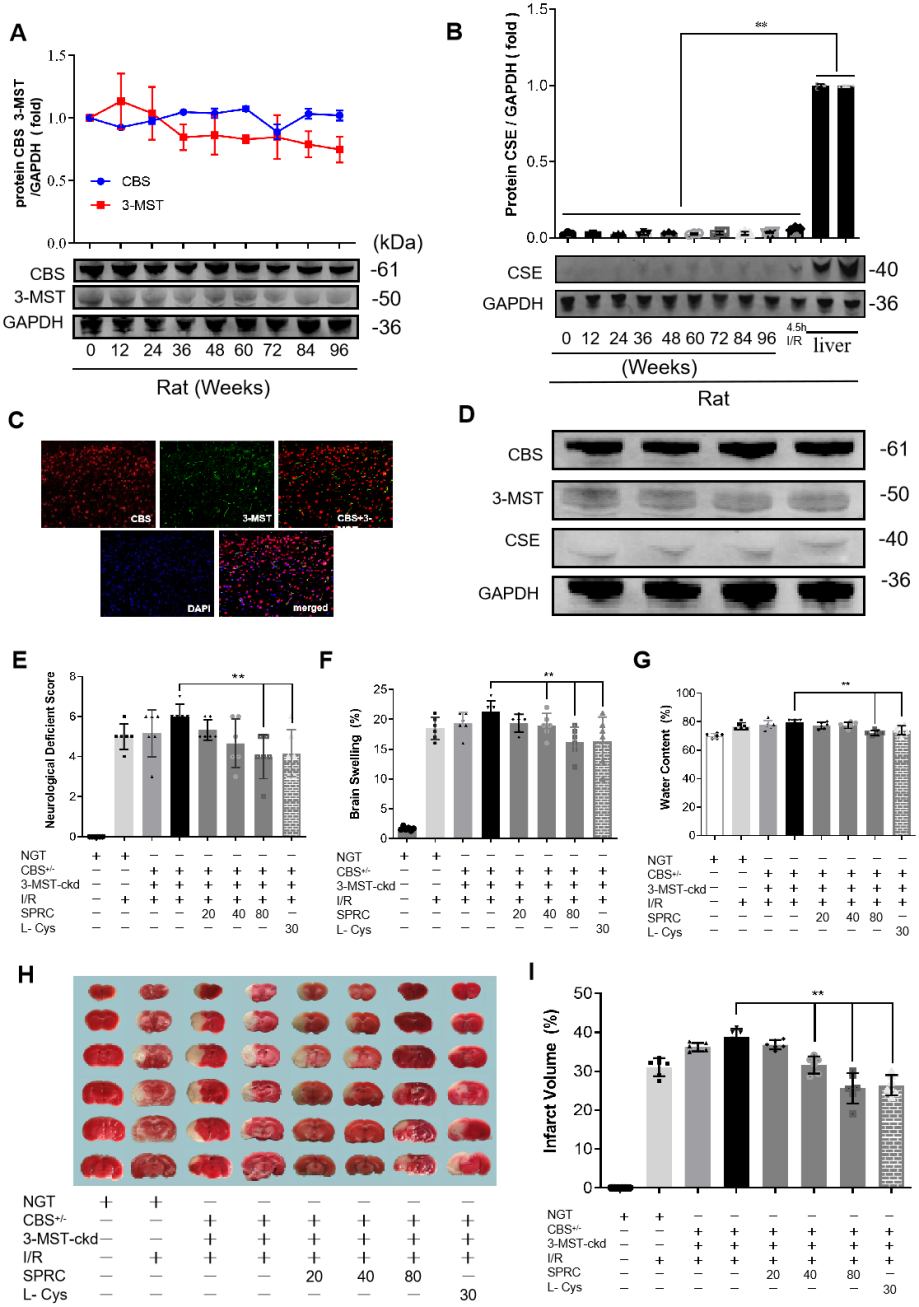
Neuroprotection of SRPC is independent of synthetase CBS and 3‐MST. (A and B) Expression pattern of CBS, 3‐MST, and CSE in the life of rats. (C) Immunofluorescent orientation of CBS and 3‐MST expression in rat brain. (D) Expression pattern of CBS, 3‐MST, and CSE in the life of mice. (E–G) Assay for neuroethology score (E), brain swelling (F), and brain water content (G) in non‐transgenic (NGT) or transgenic mice (CBS^+/−^ and CBS^+/−^ + 3‐MST cerebral knockdown mice) with/without SPRC treatment after I/R injury. (H) TTC staining for brain tissues harvested from mice in different groups. (I) Statistical data of TTC staining presented as infarct volume. The dosing units for SPRC and L‐cys are mg/kg. Data are presented as mean ± sem, one‐way ANOVA. Compared to the group CBS^+/−^ + 3‐MST‐ckd mice with I/R, **p < 0.001.

As above mentioned, in mammals, endogenous H_2_S is mainly produced by three synthetases: cystathionine‐γ‐lyase (CSE), cystathionine‐β‐synthase (CBS) and 3‐mercaptopyruvat sulfurtransferase (3‐MST). To once again confirm that CBS and 3‐MST are the two main synthetases in the brain to biosynthesize H_2_S, we detected their protein expression as well as another synthetase CSE in the lifetime of rats and mice. Our results showed that CBS and 3‐MST were indeed expressed stably in the life (from newborn to 96 weeks) of rats (Figure 6A), but conditions were opposite regarding the expression of CSE, though slight upregulated expression was found after 4.5 h I/R injury (Figure 6B). Similar results were verified in mice (Figure 6D). Besides, only CBS and 3‐MST were successfully located by immunofluorescence in rat brain tissues (Figure 6C). These data from Figure 6A through D just demonstrated that CBS and 3‐MST are indeed the two main synthetases that produce H_2_S in the brain.

Since SPRC is a putative endogenous H_2_S modulator, based on the above results, we next intended to test whether the neuroprotective role of SPRC is dependent on CBS and 3‐MST. Therefore, we evaluated the role of SPRC in CBS and/or 3‐MST transgenic mice with I/R injury. As the results showed, compared with the non‐transgenic mice with I/R injury, the neurological score, brain swelling, and water content of CBS^+/−^ mice suffering from I/R slightly increased, and conditions were much more serious in CBS^+/−^ + 3‐MST‐cerebral knock down (CBS^+/−^ + 3‐MST‐ckd) mice after I/R because these indicators were further significantly heightened. However, SPRC treatment could still greatly degrade these damage parameters in CBS^+/−^ + 3‐MST‐ckd mice (**p < 0.01). In TTC staining, similar results were observed that SPRC significantly reduced the infarct volume compared with the CBS^+/−^ + 3‐MST‐ckd I/R group (**p < 0.01). These data suggested that knockdown of H_2_S synthetases CBS and 3‐MST could aggravate cerebral I/R injury, and the neuroprotective role of SPRC seemed independent of the synthase activity of CBS and 3‐MST.

### 3.8 SPRC Protects Cerebral I/R Injury via Preserving the Endogenous Level of H_2_S

**FIGURE 7:**
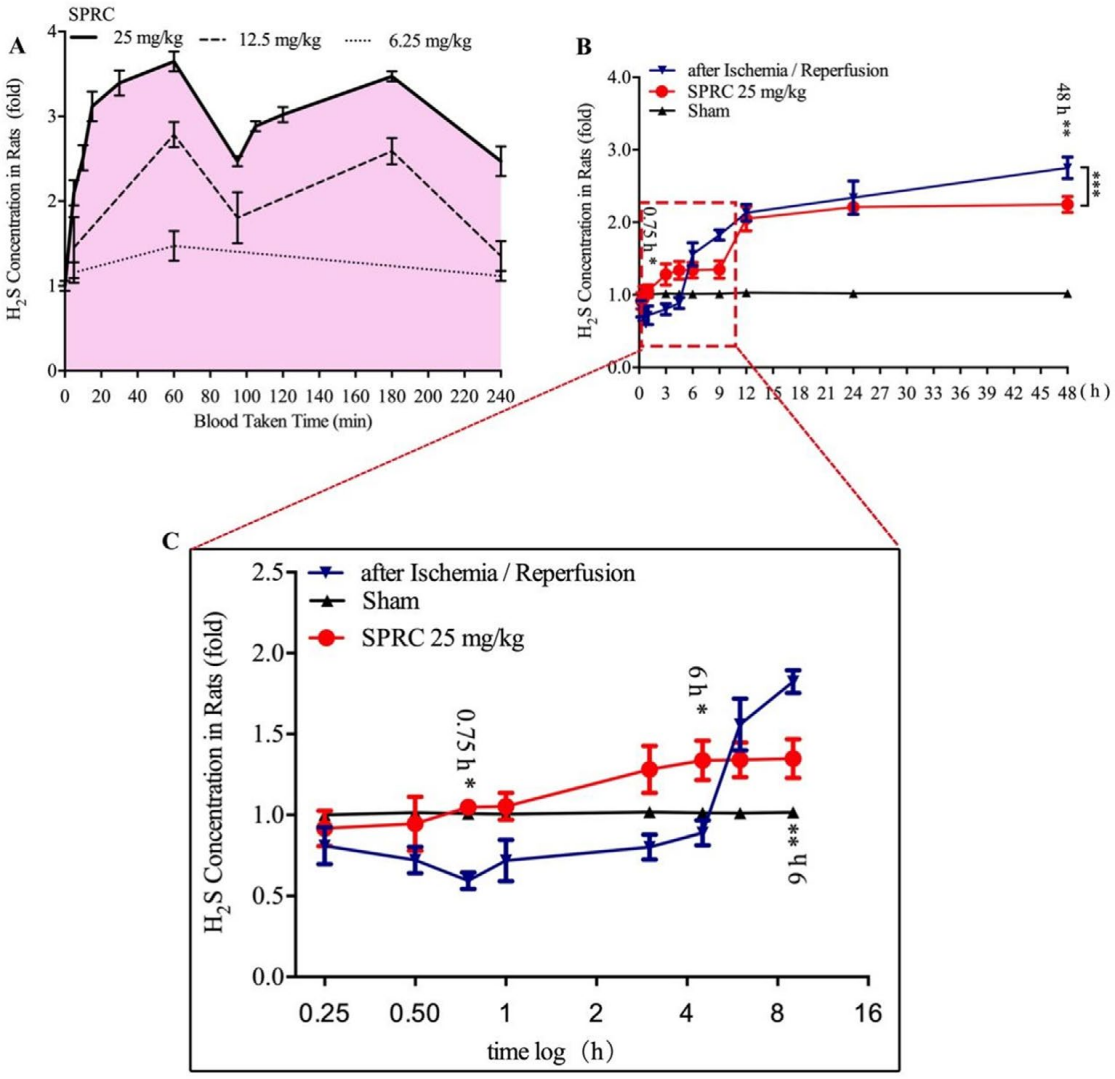
SPRC regulates endogenous H_2_S through increasing H_2_S concentration. Serum H_2_S adopted at different time points from normal or I/R rats was detected and assayed. (A) H_2_S level after SPRC administration in normal rats. (B) H_2_S concentration with/without SPRC treatment in I/R rats. (C) Enlarged graph about H_2_S level in the first 9 h after I/R in rats. Data are presented as mean ± sem; differences between two groups were analyzed using One‐way ANOVA with Tukey's HSD. Compared with I/R group at defined time points, *p < 0.05, **p < 0.01; SPRC vs I/R group, ***p < 0.001.

Since CBS and 3‐MST were dispensable for the neuroprotective role of SPRC, we doubted whether SPRC could still improve the level of H_2_S to exert its neuroprotective effects. Therefore, we next detected the H_2_S level after SPRC administration in normal rats. Results in Figure 7A manifested that serum H_2_S level rapidly increased in a dosage‐dependent manner after SPRC administration, and the concentration peak was reached 1 h later after SPRC administration. Meanwhile, the H_2_S level was also assayed in I/R rats with/without SPRC treatment. The results showed that the H_2_S level in I/R rats was significantly decreased in the first 6 h compared to that in the sham group; while the H_2_S concentration surprisingly presented a gradual increase 6 h after I/R (Figure 7B,C). However, after SPRC treatment, the H_2_S level could be upregulated in the first 6 h and then downregulated after 6 h (Figure 7B,C, *p < 0.05, **p < 0.01, ***p < 0.001). These data preliminarily demonstrated that SPRC could preserve the H_2_S level after I/R probably through direct H_2_S releasing.

### 3.9 SPRC Promotes Protective Autophagy to Block Neurological Injury

**FIGURE 8:**
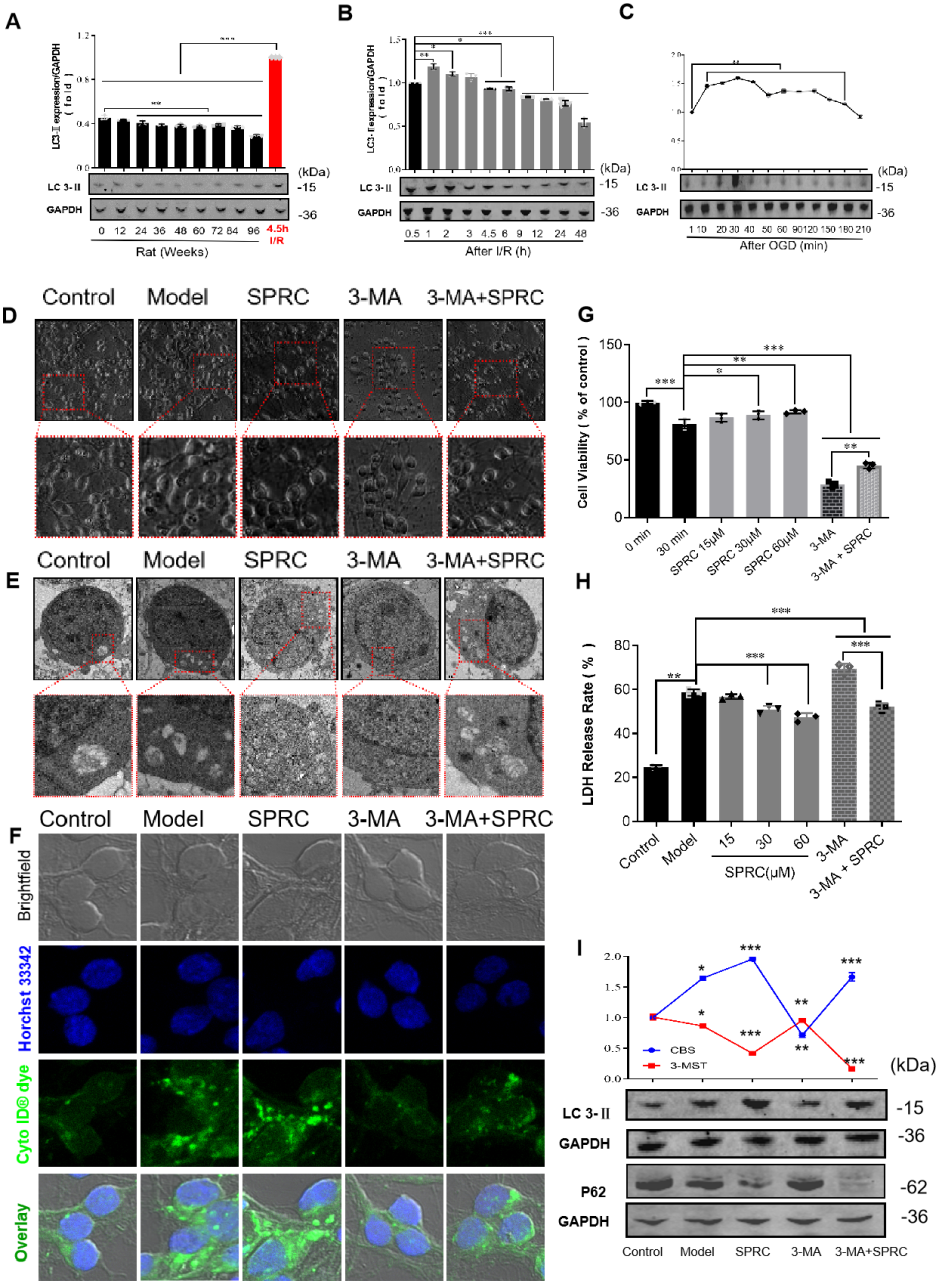
SPRC promotes protective autophagy to block neurological injury. Primary neurons were dealt with 30 min oxygen–glucose deprivation (OGD) before reoxygenation and SPRC/3‐MA (3‐methyladenine) treatment. (A) Expression pattern of autophagy marker LC 3‐II in the life of rats. (B) Activated autophagy at different time points after I/R injury in the rat brain. (C) Activated autophagy at different time points after OGD in primary neurons. (D) Morphological change of neurons under optical microscope; Scale bars: 10× (above), 40× (below). (E) Morphological changes of neurons observed by transmission electron microscopy; Scale bars: 1 μm (above), 0.5 μm (below). (F) Immunofluorescent observation of autophagy in neurons in different groups; Scale bar: 40×. (G) Cell viability of neurons after SPRC and/or 3‐MA treatment. (H) LDH release of neurons after SPRC and/or 3‐MA treatment. (I) LC 3‐II and p62 expression in neurons after OGD with SPRC and/or 3‐MA treatment. Data are presented as mean ± sem, one‐way ANOVA. Compared to defined group, *p < 0.05, **p < 0.01, ***p < 0.001.

Autophagy has been demonstrated as an important pathophysiological factor of ischemic stroke [30]. In order to explore the cellular and molecular mechanisms under the neuroprotective role of SPRC, we tried to assay the impact of SPRC on autophagy. LC 3‐II is a typical hallmark of autophagy; results showed that the expression of LC 3‐II was gradually decreased with the aging process under physiological conditions (Figure 8A, *p < 0.05), but could be sharply upregulated after rat I/R injury and oxygen glucose deprivation (OGD) in neurons (Figure 8B,C, *p < 0.05, **p < 0.01 and ***p < 0.001), implying that autophagy may play a role as a guard against ischemic stroke in our research. The following data in Figure 8G,H further verified that autophagy was protective after ischemic injury, as after inhibition of autophagy by 3‐MA, cell viability was remarkably weakened while LDH release was strongly increased after OGD injury of neurons (compared with the control group, **p < 0.01, ***p < 0.001). Furthermore, SPRC treatment could significantly improve cell viability and block LDH release after neuron OGD injury (compared to the model, *p < 0.05, **p < 0.01, ***p < 0.001). Although neuronal injury was exacerbated, presented by lower cell viability and much more LDH release after autophagy inhibition (Figure 8G,H, ***p < 0.001), SPRC was still able to alleviate neuronal injury compared to the 3‐MA group (**p < 0.01, ***p < 0.001). Additionally, in morphology, after OGD, neurons were observed shrinking and lacking synapses under the optical microscope (Figure 8D), and autophagosomes were apparently activated and increased under the transmission electron microscope and fluorescent microscope (Figure 8E,F). However, SPRC could mitigate these adverse morphological changes and improve autophagy activation, whether autophagy was inhibited by 3‐MA or not (Figure 8D through F). Finally, results of protein expression showed that SPRC indeed can further increase the expression of LC3‐II and decrease the expression of p62, though autophagy was inhibited by 3‐MA (Figure 8I, *p < 0.05, ***p < 0.001).

## 4 Discussion

The single‐cell dataset from this research sheds light on the intercellular communication network involved in stroke pathogenesis, highlighting the disrupted expression in CNS cells and the distinctive pattern of hydrogen sulfide and autophagy genes within the CNS. Utilizing this evidence, we categorized the 58,505 cells accordingly. Histologically, it was observed that the percentage of cells expressing H_2_S related genes in stroke—affected brain tissues was lower compared to those in healthy brain samples. Our findings reveal dysregulation in the expression of hydrogen sulfide and autophagy related genes, particularly in the central nervous system, such as microglial cells, astrocytes, glial cells, and neurons. Moreover, the significant increase in correlation between hydrogen sulfide and autophagy levels was greater in neurons of stroke compared to the control group. Thus, we have delivered an in‐depth single‐cell RNA sequencing analysis that clarifies the roles of H_2_S and autophagy in brain tissues affected by stroke, thereby enhancing our understanding of the CNS microenvironment and the cellular components linked to these processes.

Ischemic stroke is a kind of cerebrovascular disease and tends to attack elderly people for their decrepit and frail cerebrovascular. It seems that stroke‐related risk factors such as Hcy and ROS are elevated along with aging [51, 52, 53, 54, 55, 56]. Actually, we indeed found a gradual increase of Hcy and ROS with aging no matter in people or in animals (Tables 1 and 2). On the other hand, the gasotransmitter H_2_S has been seen as a neuromodulator though controversy exists about its role in ischemic stroke. Based on this point and the fact that the H_2_S level was also increased with aging (Tables 1 and 2), we doubt whether H_2_S might be another stroke‐related risk factor like Hcy and ROS. However, our results were surprising since only Hcy and ROS were still increased in stroke patients and cerebral I/R rats, but the H_2_S level was not (Figure 3). These results reminded us that H_2_S may be a neuroprotectant rather than a related risk factor in ischemic stroke because only a protectant could be exhausted and decreased after neuronal ischemic injury.

Since H_2_S was a possible neuroprotectant in ischemic stroke in our research, we next observed whether neuronal ischemic injury could be relieved after exogenous administration of the H_2_S donor. SPRC is a derivative from L‐cysteine synthesized by our lab, and its cytoprotective role has been defined in ischemic heart injury and heart failure [25, 29, 56, 57]. However, in the nervous system, although the anti‐inflammation of SPRC in neurological learning and memory impairment is verified [26, 27], its role in ischemic stroke still remains ambiguous. In our work, we first demonstrated that SPRC administration could ameliorate cerebral I/R injury in aspects of neurobehavioral function, neurological metabolism, and histopathological changes (Figures 6 and 7). Meanwhile, defined neurological protection was also ascertained in primary neurons suffering from OGD (Figure 8G through E). Considering that SPRC is an effective donor for H_2_S, the neuroprotective role of H_2_S in ischemic stroke was further confirmed in our research.

In the nervous system, it is reported that bioenzymatic synthesis of H_2_S is mainly dependent on CBS and 3‐MST rather than CSE [7, 8]. In this work, we did find only CBS and 3‐MST were abundantly expressed, but CSE expression was very low, no matter in rat or mouse brain tissues (Figure 6A through D). In our previous studies, the protection and anti‐inflammation potential of SPRC is dependent on its main synthetase CSE in heart injury and rheumatoid arthritis, and after inhibition of CSE, the positive potential of SPRC is abolished [25, 28, 56, 57]. However, in the present study, what is surprising is that SPRC could still mitigate neurological injury in cerebral I/R injury mice after CBS and 3‐MST knockdown (Figure 6E through I), suggesting neuroprotection of SPRC in ischemic cerebral injury was not completely dependent on its main synthetases CBS and 3‐MST. These data firstly revealed that H_2_S synthetases are dispensable for SPRC in neuroprotection against ischemic stroke.

Though the neuroprotection of SPRC in ischemic stroke was independent of the classical enzymatic pathway, it is undoubtedly true that the H_2_S level was dose‐dependently increased after SPRC administration (Figure 7). In this case, we speculated that SPRC might act through direct H_2_S release or another non‐classical enzymatic pathway to salvage the disastrously decreased H_2_S after ischemic stroke. In addition, it was interesting to note that the H_2_S level decreased in the first 6 h after I/R and then sharply increased, while this tendency could be reversed after SPRC administration (Figure 7B,C). The explanation might be: (1) in the first 6 h after I/R, a number of injured and dying neurons exhausted the balanced H_2_S pool and thus resulted in H_2_S level decrease; (2) and then 6 h later, numerous neurons died and released their stored H_2_S, thus leading to a sharp increase in H_2_S level; (3) SPRC administration could compensate for and salvage the injured and dying neurons with sufficient H_2_S through a non‐classical enzymatic pathway; as a result, dying neurons were decreased and the later phase sharply increased H_2_S was blocked. Summarily, our results first found that SPRC could preserve the endogenous balance of H_2_S levels.

In ischemic stroke, cellular autophagy has been recognized as a “double‐edged sword” and its role is still not completely conclusive [30]. It seems moderately activated autophagy plays neuroprotective role while excessive autophagy activation can generate a secondary injury to cells [58, 59]. In baseline conditions, autophagy is slightly activated and could be decreased with aging in several animal models [60]. Similarly, in our study, we found that slightly activated autophagy existed and was deceased with aging (Figure 8A). Furthermore, after ischemic injury, autophagy was strongly activated in animals and primary neurons (Figure 8B,C), and after inhibition by 3‐MA, a selective autophagy and phosphoinositide 3‐kinase inhibitor, neurological cellular injury was deteriorated (Figure 8G,E), suggesting autophagy plays a positive role in our research. On the other hand, SPRC administration could further upregulate autophagy and alleviated neuronal injury (Figure 8E,I). Interestingly, even though inhibited by 3‐MA, autophagy was still upregulated by SPRC administration, demonstrating that SPRC could upregulate the baseline level of autophagy. In addition, SPRC led to no p62 band (Figure 8I). This could be due to the complex interplay between autophagy and other cellular processes that are affected by the combination of 3‐MA and SPRC. As is well known 3‐Methyladenine (3‐MA) is an autophagy inhibitor that blocks the formation of autophagosome precursors by inhibiting the activity of the class III PI3K, Vps34 [61, 62, 63]. When autophagy is inhibited by 3‐MA, the degradation of p62 via the autophagy‐lysosome pathway is also impeded [64]. As a result, p62 accumulates in the cell. However, in the presence of SPRC, it is possible that the combined effect of 3‐MA and SPRC leads to a scenario where p62 is either being actively degraded through an alternative pathway or is not being synthesized at a detectable level, thus resulting in no visible p62 band.

All in all, we firstly revealed that SPRC could promote protective autophagy to realize its neuroprotection potential. However, further exploration of more detailed molecular mechanisms is still needed to launch.

In interpreting the findings of SPRC on MCAO in animals, it is important to consider certain limitations. Firstly, the neurological behavior assays used in our study provide valuable insights, but they may not capture the full spectrum of neurological functions affected by stroke. Secondly, the dosage and treatment regimen of SPRC used in our study may not be optimized for all potential applications, and the long‐term effects of SPRC treatment were not assessed. Furthermore, while we observed an upregulation of protective autophagy with SPRC administration, the precise signaling pathways and the duration of this effect are not fully understood.

## 5 Conclusions

Based on the present data from our study, we herein firstly concluded that: (1) CNS cell dysregulation of hydrogen sulfide and autophagy genes is prominent, with neurons in stroke patients displaying a significant rise in their interrelation compared to controls. (2) SPRC, a H_2_S donor, could protect against ischemic stroke independent of the classical enzymatic CBS/3‐MST pathway; (3) Promotion of SPRC to the protective autophagy activation is involved in the cellular mechanisms of its neuroprotection in cerebral I/R injury.

## Author Contributions

X.X. and L.M.: Writing – original draft; L.C. and Y.W. analyzed the data; Z.Z. and L.M. analyzed the data; J.Q. and Y.M. revised the paper; and YZ.Z.: Supervision, writing – review and editing.

## Conflicts of Interest

The authors declare no conflicts of interest.

## Supporting information


Data S1.

Data S2.

Table S1.

## Data Availability

The data that support the findings of this study are available from the corresponding author upon reasonable request.
