# Supplementary material for: S‐Propargyl‐Cysteine Attenuates Stroke Heterogeneity via Promoting Protective Autophagy Across Multiple Neural Cell Types: Insights From Single‐Cell Sequencing
Source: CNS Neurosci Ther. 2025 Jul 24;31(7):e70399. doi: 10.1111/cns.70399 (PMC12287620; doi:10.1111/cns.70399)
Supplement: Supplementary file 1 — Data S1. [file CNS-31-e70399-s002.pdf]

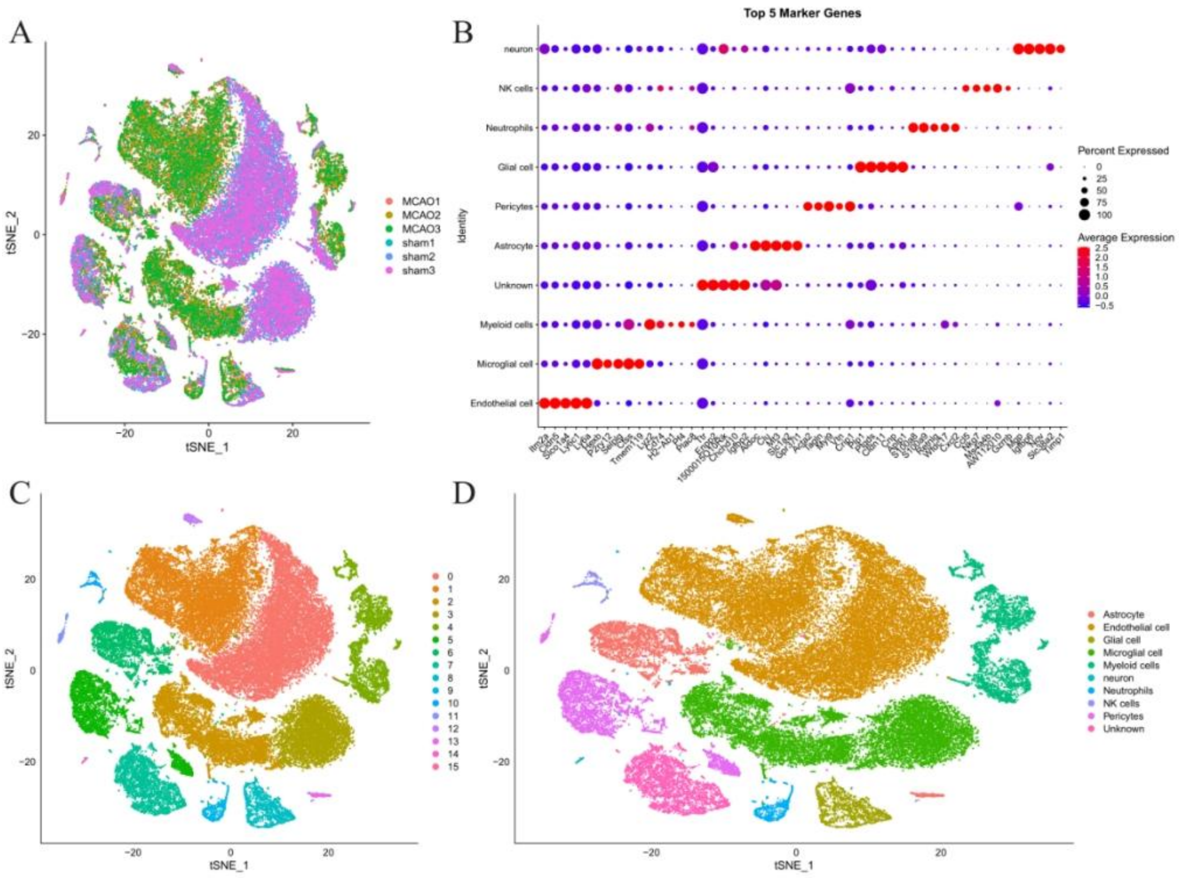

SUPPLEMENTARY FIGURE 1. Preprocessing of scRNA-seq data for downstream analysis. (A) The dispersion of samples post-dataset integration via the harmony algorithm was depicted through t-SNE plots. (B) A heatmap was constructed to illustrate the expression profiles of signature genes within the detected cell populations. (C) Utilizing a clustering technique set at a resolution of 0.1, 16 distinct clusters were delineated. (D) A t-SNE plot was employed to represent the distribution across the nine varied cell types.

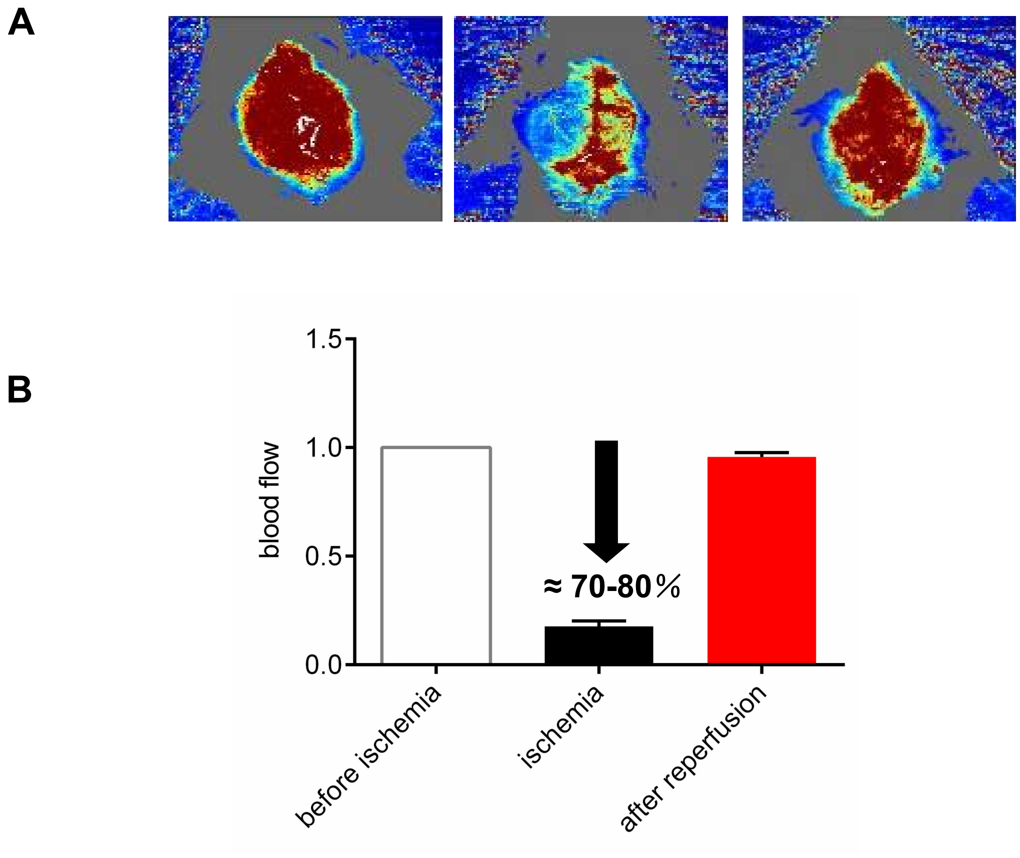

SUPPLEMENTARY FIGURE 2. Doppler monitoring in the MCAO model preparation. (A) Representative images of CBF; (B) Cerebral blood flow rate analyis after ischemia.

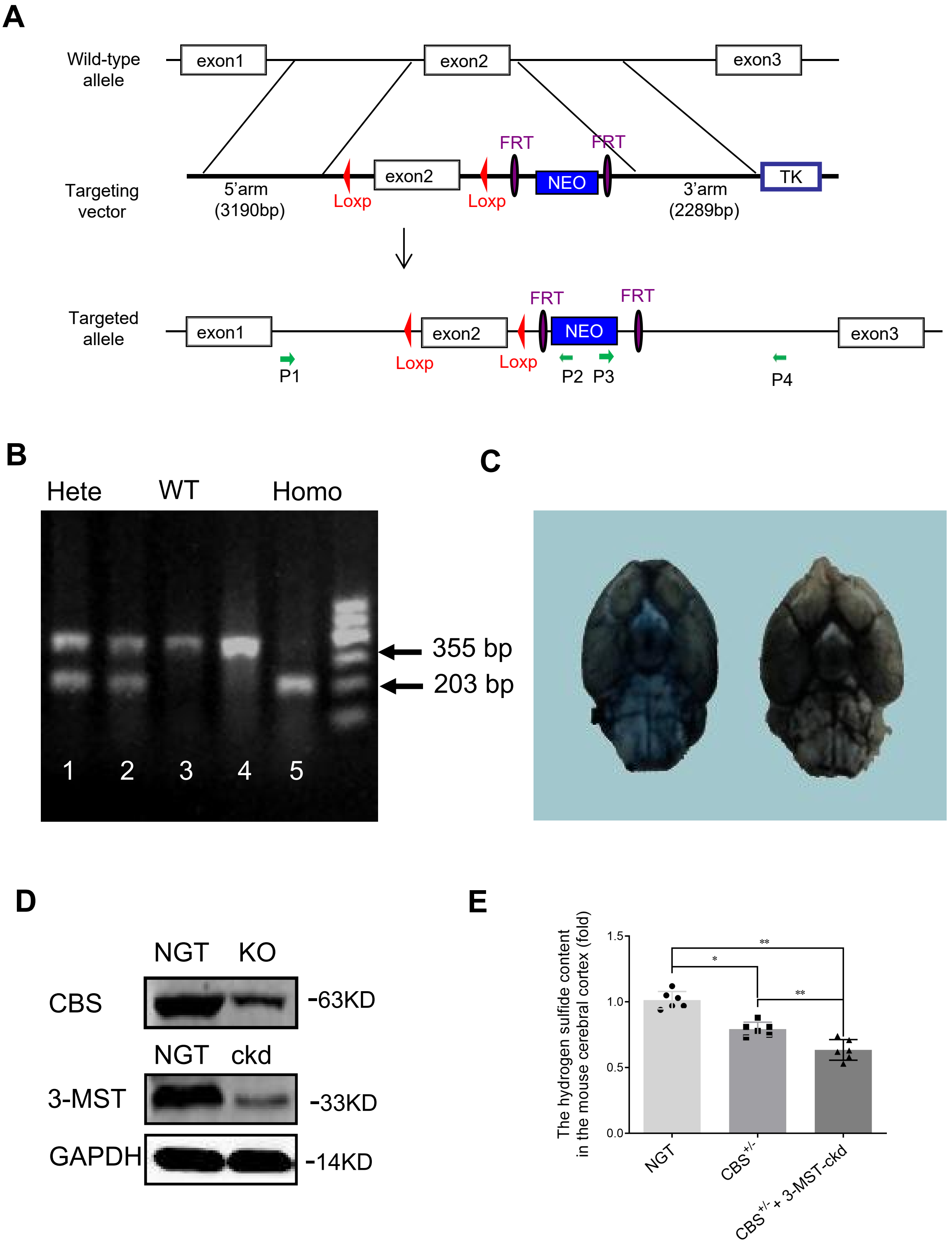

SUPPLEMENTARY FIGURE 3. Establishment and accessment of animal model for CBS and CBS$^{+/-}$+ 3-MST-ckd mouse.(A) The production method of CBS knockout mice;(B) gene identification of CBS knockout mice; (C) Anatomy of blood vessels in the skull base of CBS knockout mice; (D) The expression of CBS knockout mice and CBS$^{+/-}$+ 3-MST-ckd; (E) The hydrogen sulfide content in the cerebral cortex of CBS$^{+/-}$+ 3-MST-ckd mouse.

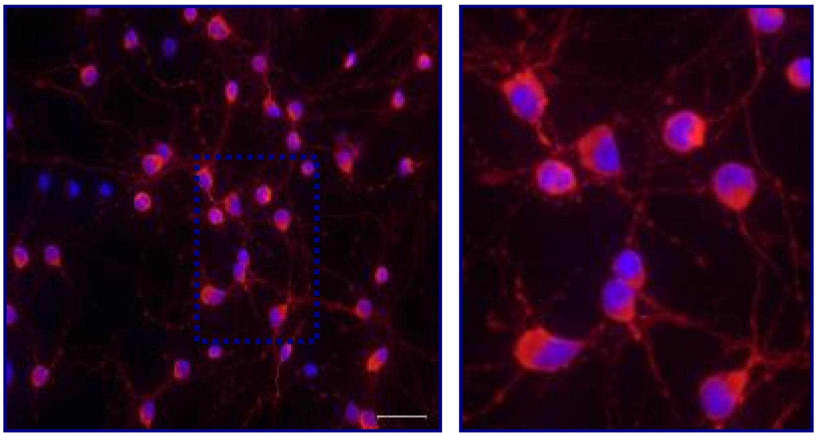

> 95%

SUPPLEMENTARY FIGURE 4. Identification of Primary Neurons from Rats

SUPPLEMENTARY TABLE 1. CBS$^{+/-}$、3-MST-ckd Physiological parameters

	WT	NTG	CBS$^{-/+}$	CBS$^{-/+}$ + 3-MST-ckd
pH	7.35±0.18	7.36±0.15	7.34±0.24	7.35±0.13
pO_2 (mm Hg)	117.30±9.77	112.25±17.43	109.55±11.41	111.00±13.41
pCO_2 (mm Hg)	35.34±10.51	31.35±9.97	30.37±9.78	31.44±12.56
DBP (mm Hg)	109.81±10.56	115.33±9.74	114.65±12.83	110.41±13.90
SBP (mm Hg)	148.58±17.45	144.98±9.09	142.4±10.09	147.62±15.47
Heart rate (bpm)	569.71±72.83	498.2±107.71	529.5±97.35	544.26±96.52
Body weight (g)	25.16±1.10	25.69±1.58	26.69±1.13	27.30±1.24
