# Supplementary material for: S‐Propargyl‐Cysteine Attenuates Stroke Heterogeneity via Promoting Protective Autophagy Across Multiple Neural Cell Types: Insights From Single‐Cell Sequencing
Source: CNS Neurosci Ther. 2025 Jul 24;31(7):e70399. doi: 10.1111/cns.70399 (PMC12287620; doi:10.1111/cns.70399)
Supplement: Supplementary file 2 — Data S2. [file CNS-31-e70399-s003.docx]

Inclusion criteria for blood sample collection and testing in stroke patients are usually as follows:

**I. Clinical diagnostic criteria**

1.Definite stroke diagnosis

Patients must be diagnosed as stroke through clinical evaluation, including medical history collection, neurological examination and imaging examinations (such as head CT or MRI), including ischemic stroke or hemorrhagic stroke.

2.Onset time

Generally, it is required to be within a specific onset time range, such as within several hours to several days after onset. The specific time depends on the research purpose and the characteristics of blood sample detection indicators.

**II. Age and gender requirements**

1.Age range

May be limited to a different special age groups, or include patients of different age groups according to research needs.

2.Gender

Some studies may have specific requirements for gender, or include both male and female patients to ensure the universality of research results.

**III. Conditions related to exclusion criteria**

1.Other serious diseases

Patients with severe diseases of important organs such as heart, liver and kidney are excluded, because these diseases may affect the results of blood sample testing.

2.Recent surgery or trauma

Patients with major surgery or severe trauma in the recent period (such as within one month) are usually excluded to avoid the interference of surgery or trauma on blood sample indicators.

3.Hematological diseases

Patients with hematological diseases (such as leukemia, hemophilia, etc.) are not suitable for inclusion, because these diseases will affect blood components and test results.

4.Pregnant or lactating women

To protect the health of mothers and infants, pregnant or lactating women are generally not included in the study.

**IV. Informed consent**

1. Patients or their legal representatives must fully understand the purpose, methods, risks and benefits of the study and sign an informed consent form.
