# Supplementary material for: S‐Propargyl‐Cysteine Attenuates Stroke Heterogeneity via Promoting Protective Autophagy Across Multiple Neural Cell Types: Insights From Single‐Cell Sequencing
Source: CNS Neurosci Ther. 2025 Jul 24;31(7):e70399. doi: 10.1111/cns.70399 (PMC12287620; doi:10.1111/cns.70399)
Supplement: Supplementary file 3 — Table S1. [file CNS-31-e70399-s001.docx]

| ID | Gene Symbol | GSEA_name | Brief description |
| --- | --- | --- | --- |
| 1 | Cth | MM9615 | The chemical reactions and pathways involving hydrogen sulfide, H2S. |
| 2 | Cbs | MM9615 | The chemical reactions and pathways involving hydrogen sulfide, H2S. |
| 3 | Sp1 | MM9615 | The chemical reactions and pathways involving hydrogen sulfide, H2S. |
| 4 | Mpst | MM9615 | The chemical reactions and pathways involving hydrogen sulfide, H2S. |
| 5 | Ethe1 | MM9615 | The chemical reactions and pathways involving hydrogen sulfide, H2S. |
| 6 | Cth | MM13529 | Catalysis of the elimination of hydrogen sulfide or substituted H2S. |
| 7 | Glo1 | MM13529 | Catalysis of the elimination of hydrogen sulfide or substituted H2S. |
| 8 | Alox5ap | MM13529 | Catalysis of the elimination of hydrogen sulfide or substituted H2S. |
| 9 | Gstm4 | MM13529 | Catalysis of the elimination of hydrogen sulfide or substituted H2S. |
| 10 | Hccs | MM13529 | Catalysis of the elimination of hydrogen sulfide or substituted H2S. |
| 11 | Ltc4s | MM13529 | Catalysis of the elimination of hydrogen sulfide or substituted H2S. |
| 12 | Mgst2 | MM13529 | Catalysis of the elimination of hydrogen sulfide or substituted H2S. |
| 13 | Kyat3 | MM13529 | Catalysis of the elimination of hydrogen sulfide or substituted H2S. |
| 14 | Scly | MM13529 | Catalysis of the elimination of hydrogen sulfide or substituted H2S. |
| 15 | Mgst3 | MM13529 | Catalysis of the elimination of hydrogen sulfide or substituted H2S. |
| 16 | Kyat1 | MM13529 | Catalysis of the elimination of hydrogen sulfide or substituted H2S. |
| 17 | Cenpv | MM13529 | Catalysis of the elimination of hydrogen sulfide or substituted H2S. |

| ID | Gene Symbol | GSEA_name | Brief description |
| --- | --- | --- | --- |
| 1 | Trim68 | MM5430 | Any process that activates, maintains or increases the rate of autophagy. Autophagy is the process in which cells digest parts of their own cytoplasm. |
| 2 | Prkaa1 | MM5430 | Any process that activates, maintains or increases the rate of autophagy. Autophagy is the process in which cells digest parts of their own cytoplasm. |
| 3 | Ticam1 | MM5430 | Any process that activates, maintains or increases the rate of autophagy. Autophagy is the process in which cells digest parts of their own cytoplasm. |
| 4 | Nod1 | MM5430 | Any process that activates, maintains or increases the rate of autophagy. Autophagy is the process in which cells digest parts of their own cytoplasm. |
| 5 | Prkaa2 | MM5430 | Any process that activates, maintains or increases the rate of autophagy. Autophagy is the process in which cells digest parts of their own cytoplasm. |
| 6 | Pip4k2b | MM5430 | Any process that activates, maintains or increases the rate of autophagy. Autophagy is the process in which cells digest parts of their own cytoplasm. |
| 7 | Adrb2 | MM5430 | Any process that activates, maintains or increases the rate of autophagy. Autophagy is the process in which cells digest parts of their own cytoplasm. |
| 8 | Ager | MM5430 | Any process that activates, maintains or increases the rate of autophagy. Autophagy is the process in which cells digest parts of their own cytoplasm. |
| 9 | Pip4k2c | MM5430 | Any process that activates, maintains or increases the rate of autophagy. Autophagy is the process in which cells digest parts of their own cytoplasm. |
| 10 | Slc25a4 | MM5430 | Any process that activates, maintains or increases the rate of autophagy. Autophagy is the process in which cells digest parts of their own cytoplasm. |
| 11 | Slc25a5 | MM5430 | Any process that activates, maintains or increases the rate of autophagy. Autophagy is the process in which cells digest parts of their own cytoplasm. |
| 12 | Bid | MM5430 | Any process that activates, maintains or increases the rate of autophagy. Autophagy is the process in which cells digest parts of their own cytoplasm. |
| 13 | Bcl2l11 | MM5430 | Any process that activates, maintains or increases the rate of autophagy. Autophagy is the process in which cells digest parts of their own cytoplasm. |
| 14 | Bnip3 | MM5430 | Any process that activates, maintains or increases the rate of autophagy. Autophagy is the process in which cells digest parts of their own cytoplasm. |
| 15 | Bnip3l | MM5430 | Any process that activates, maintains or increases the rate of autophagy. Autophagy is the process in which cells digest parts of their own cytoplasm. |
| 16 | Rb1cc1 | MM5430 | Any process that activates, maintains or increases the rate of autophagy. Autophagy is the process in which cells digest parts of their own cytoplasm. |
| 17 | Plk3 | MM5430 | Any process that activates, maintains or increases the rate of autophagy. Autophagy is the process in which cells digest parts of their own cytoplasm. |
| 18 | Dcn | MM5430 | Any process that activates, maintains or increases the rate of autophagy. Autophagy is the process in which cells digest parts of their own cytoplasm. |
| 19 | Ddit3 | MM5430 | Any process that activates, maintains or increases the rate of autophagy. Autophagy is the process in which cells digest parts of their own cytoplasm. |
| 20 | Endog | MM5430 | Any process that activates, maintains or increases the rate of autophagy. Autophagy is the process in which cells digest parts of their own cytoplasm. |
| 21 | Epm2a | MM5430 | Any process that activates, maintains or increases the rate of autophagy. Autophagy is the process in which cells digest parts of their own cytoplasm. |
| 22 | Sesn1 | MM5430 | Any process that activates, maintains or increases the rate of autophagy. Autophagy is the process in which cells digest parts of their own cytoplasm. |
| 23 | Gnai3 | MM5430 | Any process that activates, maintains or increases the rate of autophagy. Autophagy is the process in which cells digest parts of their own cytoplasm. |
| 24 | Htt | MM5430 | Any process that activates, maintains or increases the rate of autophagy. Autophagy is the process in which cells digest parts of their own cytoplasm. |
| 25 | Hif1a | MM5430 | Any process that activates, maintains or increases the rate of autophagy. Autophagy is the process in which cells digest parts of their own cytoplasm. |
| 26 | Hmgb1 | MM5430 | Any process that activates, maintains or increases the rate of autophagy. Autophagy is the process in which cells digest parts of their own cytoplasm. |
| 27 | Hmox1 | MM5430 | Any process that activates, maintains or increases the rate of autophagy. Autophagy is the process in which cells digest parts of their own cytoplasm. |
| 28 | Irgm1 | MM5430 | Any process that activates, maintains or increases the rate of autophagy. Autophagy is the process in which cells digest parts of their own cytoplasm. |
| 29 | Ifnb1 | MM5430 | Any process that activates, maintains or increases the rate of autophagy. Autophagy is the process in which cells digest parts of their own cytoplasm. |
| 30 | Ifng | MM5430 | Any process that activates, maintains or increases the rate of autophagy. Autophagy is the process in which cells digest parts of their own cytoplasm. |
| 31 | Igtp | MM5430 | Any process that activates, maintains or increases the rate of autophagy. Autophagy is the process in which cells digest parts of their own cytoplasm. |
| 32 | Ikbkg | MM5430 | Any process that activates, maintains or increases the rate of autophagy. Autophagy is the process in which cells digest parts of their own cytoplasm. |
| 33 | Il4 | MM5430 | Any process that activates, maintains or increases the rate of autophagy. Autophagy is the process in which cells digest parts of their own cytoplasm. |
| 34 | Kdr | MM5430 | Any process that activates, maintains or increases the rate of autophagy. Autophagy is the process in which cells digest parts of their own cytoplasm. |
| 35 | Snx18 | MM5430 | Any process that activates, maintains or increases the rate of autophagy. Autophagy is the process in which cells digest parts of their own cytoplasm. |
| 36 | Nprl3 | MM5430 | Any process that activates, maintains or increases the rate of autophagy. Autophagy is the process in which cells digest parts of their own cytoplasm. |
| 37 | Fyco1 | MM5430 | Any process that activates, maintains or increases the rate of autophagy. Autophagy is the process in which cells digest parts of their own cytoplasm. |
| 38 | Sqstm1 | MM5430 | Any process that activates, maintains or increases the rate of autophagy. Autophagy is the process in which cells digest parts of their own cytoplasm. |
| 39 | Pafah1b2 | MM5430 | Any process that activates, maintains or increases the rate of autophagy. Autophagy is the process in which cells digest parts of their own cytoplasm. |
| 40 | Cdk16 | MM5430 | Any process that activates, maintains or increases the rate of autophagy. Autophagy is the process in which cells digest parts of their own cytoplasm. |
| 41 | Pik3c2a | MM5430 | Any process that activates, maintains or increases the rate of autophagy. Autophagy is the process in which cells digest parts of their own cytoplasm. |
| 42 | Pim2 | MM5430 | Any process that activates, maintains or increases the rate of autophagy. Autophagy is the process in which cells digest parts of their own cytoplasm. |
| 43 | Pip4k2a | MM5430 | Any process that activates, maintains or increases the rate of autophagy. Autophagy is the process in which cells digest parts of their own cytoplasm. |
| 44 | Prkd1 | MM5430 | Any process that activates, maintains or increases the rate of autophagy. Autophagy is the process in which cells digest parts of their own cytoplasm. |
| 45 | Ripk2 | MM5430 | Any process that activates, maintains or increases the rate of autophagy. Autophagy is the process in which cells digest parts of their own cytoplasm. |
| 46 | Rfpl4 | MM5430 | Any process that activates, maintains or increases the rate of autophagy. Autophagy is the process in which cells digest parts of their own cytoplasm. |
| 47 | Rab12 | MM5430 | Any process that activates, maintains or increases the rate of autophagy. Autophagy is the process in which cells digest parts of their own cytoplasm. |
| 48 | Rock1 | MM5430 | Any process that activates, maintains or increases the rate of autophagy. Autophagy is the process in which cells digest parts of their own cytoplasm. |
| 49 | Trim30a | MM5430 | Any process that activates, maintains or increases the rate of autophagy. Autophagy is the process in which cells digest parts of their own cytoplasm. |
| 50 | Plk2 | MM5430 | Any process that activates, maintains or increases the rate of autophagy. Autophagy is the process in which cells digest parts of their own cytoplasm. |
| 51 | Sptlc2 | MM5430 | Any process that activates, maintains or increases the rate of autophagy. Autophagy is the process in which cells digest parts of their own cytoplasm. |
| 52 | Trim21 | MM5430 | Any process that activates, maintains or increases the rate of autophagy. Autophagy is the process in which cells digest parts of their own cytoplasm. |
| 53 | Stk11 | MM5430 | Any process that activates, maintains or increases the rate of autophagy. Autophagy is the process in which cells digest parts of their own cytoplasm. |
| 54 | Snx30 | MM5430 | Any process that activates, maintains or increases the rate of autophagy. Autophagy is the process in which cells digest parts of their own cytoplasm. |
| 55 | Supt5 | MM5430 | Any process that activates, maintains or increases the rate of autophagy. Autophagy is the process in which cells digest parts of their own cytoplasm. |
| 56 | Trim30d | MM5430 | Any process that activates, maintains or increases the rate of autophagy. Autophagy is the process in which cells digest parts of their own cytoplasm. |
| 57 | Trim38 | MM5430 | Any process that activates, maintains or increases the rate of autophagy. Autophagy is the process in which cells digest parts of their own cytoplasm. |
| 58 | Tfeb | MM5430 | Any process that activates, maintains or increases the rate of autophagy. Autophagy is the process in which cells digest parts of their own cytoplasm. |
| 59 | Trim58 | MM5430 | Any process that activates, maintains or increases the rate of autophagy. Autophagy is the process in which cells digest parts of their own cytoplasm. |
| 60 | Flcn | MM5430 | Any process that activates, maintains or increases the rate of autophagy. Autophagy is the process in which cells digest parts of their own cytoplasm. |
| 61 | Tom1 | MM5430 | Any process that activates, maintains or increases the rate of autophagy. Autophagy is the process in which cells digest parts of their own cytoplasm. |
| 62 | Tsc2 | MM5430 | Any process that activates, maintains or increases the rate of autophagy. Autophagy is the process in which cells digest parts of their own cytoplasm. |
| 63 | Ulk1 | MM5430 | Any process that activates, maintains or increases the rate of autophagy. Autophagy is the process in which cells digest parts of their own cytoplasm. |
| 64 | Vdac1 | MM5430 | Any process that activates, maintains or increases the rate of autophagy. Autophagy is the process in which cells digest parts of their own cytoplasm. |
| 65 | Xbp1 | MM5430 | Any process that activates, maintains or increases the rate of autophagy. Autophagy is the process in which cells digest parts of their own cytoplasm. |
| 66 | Wac | MM5430 | Any process that activates, maintains or increases the rate of autophagy. Autophagy is the process in which cells digest parts of their own cytoplasm. |
| 67 | Rab3gap1 | MM5430 | Any process that activates, maintains or increases the rate of autophagy. Autophagy is the process in which cells digest parts of their own cytoplasm. |
| 68 | Atf6 | MM5430 | Any process that activates, maintains or increases the rate of autophagy. Autophagy is the process in which cells digest parts of their own cytoplasm. |
| 69 | Lrsam1 | MM5430 | Any process that activates, maintains or increases the rate of autophagy. Autophagy is the process in which cells digest parts of their own cytoplasm. |
| 70 | Ambra1 | MM5430 | Any process that activates, maintains or increases the rate of autophagy. Autophagy is the process in which cells digest parts of their own cytoplasm. |
| 71 | Elapor1 | MM5430 | Any process that activates, maintains or increases the rate of autophagy. Autophagy is the process in which cells digest parts of their own cytoplasm. |
| 72 | Zc3h12a | MM5430 | Any process that activates, maintains or increases the rate of autophagy. Autophagy is the process in which cells digest parts of their own cytoplasm. |
| 73 | Sesn2 | MM5430 | Any process that activates, maintains or increases the rate of autophagy. Autophagy is the process in which cells digest parts of their own cytoplasm. |
| 74 | Vps13d | MM5430 | Any process that activates, maintains or increases the rate of autophagy. Autophagy is the process in which cells digest parts of their own cytoplasm. |
| 75 | Setd2 | MM5430 | Any process that activates, maintains or increases the rate of autophagy. Autophagy is the process in which cells digest parts of their own cytoplasm. |
| 76 | Smcr8 | MM5430 | Any process that activates, maintains or increases the rate of autophagy. Autophagy is the process in which cells digest parts of their own cytoplasm. |
| 77 | Mid2 | MM5430 | Any process that activates, maintains or increases the rate of autophagy. Autophagy is the process in which cells digest parts of their own cytoplasm. |
| 78 | Tlr2 | MM5430 | Any process that activates, maintains or increases the rate of autophagy. Autophagy is the process in which cells digest parts of their own cytoplasm. |
| 79 | Trim30b | MM5430 | Any process that activates, maintains or increases the rate of autophagy. Autophagy is the process in which cells digest parts of their own cytoplasm. |
| 80 | Zdhhc19 | MM5430 | Any process that activates, maintains or increases the rate of autophagy. Autophagy is the process in which cells digest parts of their own cytoplasm. |
| 81 | Tpcn1 | MM5430 | Any process that activates, maintains or increases the rate of autophagy. Autophagy is the process in which cells digest parts of their own cytoplasm. |
| 82 | Nod2 | MM5430 | Any process that activates, maintains or increases the rate of autophagy. Autophagy is the process in which cells digest parts of their own cytoplasm. |
| 83 | Map2k1 | MM5430 | Any process that activates, maintains or increases the rate of autophagy. Autophagy is the process in which cells digest parts of their own cytoplasm. |
| 84 | Map3k7 | MM5430 | Any process that activates, maintains or increases the rate of autophagy. Autophagy is the process in which cells digest parts of their own cytoplasm. |
| 85 | Mapk3 | MM5430 | Any process that activates, maintains or increases the rate of autophagy. Autophagy is the process in which cells digest parts of their own cytoplasm. |
| 86 | Sptlc1 | MM5430 | Any process that activates, maintains or increases the rate of autophagy. Autophagy is the process in which cells digest parts of their own cytoplasm. |
| 87 | Rnf31 | MM5430 | Any process that activates, maintains or increases the rate of autophagy. Autophagy is the process in which cells digest parts of their own cytoplasm. |
| 88 | Wdr24 | MM5430 | Any process that activates, maintains or increases the rate of autophagy. Autophagy is the process in which cells digest parts of their own cytoplasm. |
| 89 | Depdc5 | MM5430 | Any process that activates, maintains or increases the rate of autophagy. Autophagy is the process in which cells digest parts of their own cytoplasm. |
| 90 | Bag3 | MM5430 | Any process that activates, maintains or increases the rate of autophagy. Autophagy is the process in which cells digest parts of their own cytoplasm. |
| 91 | Ulk2 | MM5430 | Any process that activates, maintains or increases the rate of autophagy. Autophagy is the process in which cells digest parts of their own cytoplasm. |
| 92 | Trim12c | MM5430 | Any process that activates, maintains or increases the rate of autophagy. Autophagy is the process in which cells digest parts of their own cytoplasm. |
| 93 | Smo | MM5430 | Any process that activates, maintains or increases the rate of autophagy. Autophagy is the process in which cells digest parts of their own cytoplasm. |
| 94 | Rnf152 | MM5430 | Any process that activates, maintains or increases the rate of autophagy. Autophagy is the process in which cells digest parts of their own cytoplasm. |
| 95 | Atg2a | MM5430 | Any process that activates, maintains or increases the rate of autophagy. Autophagy is the process in which cells digest parts of their own cytoplasm. |
| 96 | Trim65 | MM5430 | Any process that activates, maintains or increases the rate of autophagy. Autophagy is the process in which cells digest parts of their own cytoplasm. |
| 97 | Trim34b | MM5430 | Any process that activates, maintains or increases the rate of autophagy. Autophagy is the process in which cells digest parts of their own cytoplasm. |
| 98 | Trim30c | MM5430 | Any process that activates, maintains or increases the rate of autophagy. Autophagy is the process in which cells digest parts of their own cytoplasm. |
| 99 | Rufy4 | MM5430 | Any process that activates, maintains or increases the rate of autophagy. Autophagy is the process in which cells digest parts of their own cytoplasm. |
| 100 | Prkn | MM5430 | Any process that activates, maintains or increases the rate of autophagy. Autophagy is the process in which cells digest parts of their own cytoplasm. |
| 101 | Atg13 | MM5430 | Any process that activates, maintains or increases the rate of autophagy. Autophagy is the process in which cells digest parts of their own cytoplasm. |
| 102 | Wipi1 | MM5430 | Any process that activates, maintains or increases the rate of autophagy. Autophagy is the process in which cells digest parts of their own cytoplasm. |
| 103 | Irgm2 | MM5430 | Any process that activates, maintains or increases the rate of autophagy. Autophagy is the process in which cells digest parts of their own cytoplasm. |
| 104 | Mefv | MM5430 | Any process that activates, maintains or increases the rate of autophagy. Autophagy is the process in which cells digest parts of their own cytoplasm. |
| 105 | Wdr45 | MM5430 | Any process that activates, maintains or increases the rate of autophagy. Autophagy is the process in which cells digest parts of their own cytoplasm. |
| 106 | Sh3glb1 | MM5430 | Any process that activates, maintains or increases the rate of autophagy. Autophagy is the process in which cells digest parts of their own cytoplasm. |
| 107 | Nprl2 | MM5430 | Any process that activates, maintains or increases the rate of autophagy. Autophagy is the process in which cells digest parts of their own cytoplasm. |
| 108 | Becn1 | MM5430 | Any process that activates, maintains or increases the rate of autophagy. Autophagy is the process in which cells digest parts of their own cytoplasm. |
| 109 | Scoc | MM5430 | Any process that activates, maintains or increases the rate of autophagy. Autophagy is the process in which cells digest parts of their own cytoplasm. |
| 110 | Tmem59 | MM5430 | Any process that activates, maintains or increases the rate of autophagy. Autophagy is the process in which cells digest parts of their own cytoplasm. |
| 111 | Foxo1 | MM5430 | Any process that activates, maintains or increases the rate of autophagy. Autophagy is the process in which cells digest parts of their own cytoplasm. |
| 112 | Tbk1 | MM5430 | Any process that activates, maintains or increases the rate of autophagy. Autophagy is the process in which cells digest parts of their own cytoplasm. |
| 113 | Foxo3 | MM5430 | Any process that activates, maintains or increases the rate of autophagy. Autophagy is the process in which cells digest parts of their own cytoplasm. |
| 114 | Gsk3b | MM5430 | Any process that activates, maintains or increases the rate of autophagy. Autophagy is the process in which cells digest parts of their own cytoplasm. |
| 115 | Trp53inp1 | MM5430 | Any process that activates, maintains or increases the rate of autophagy. Autophagy is the process in which cells digest parts of their own cytoplasm. |
| 116 | Gsk3a | MM5430 | Any process that activates, maintains or increases the rate of autophagy. Autophagy is the process in which cells digest parts of their own cytoplasm. |
| 117 | Moap1 | MM5430 | Any process that activates, maintains or increases the rate of autophagy. Autophagy is the process in which cells digest parts of their own cytoplasm. |
| 118 | Ralb | MM5430 | Any process that activates, maintains or increases the rate of autophagy. Autophagy is the process in which cells digest parts of their own cytoplasm. |
| 119 | Tsc1 | MM5430 | Any process that activates, maintains or increases the rate of autophagy. Autophagy is the process in which cells digest parts of their own cytoplasm. |
| 120 | Trim13 | MM5430 | Any process that activates, maintains or increases the rate of autophagy. Autophagy is the process in which cells digest parts of their own cytoplasm. |
| 121 | Ormdl3 | MM5430 | Any process that activates, maintains or increases the rate of autophagy. Autophagy is the process in which cells digest parts of their own cytoplasm. |
| 122 | Lrrk2 | MM5430 | Any process that activates, maintains or increases the rate of autophagy. Autophagy is the process in which cells digest parts of their own cytoplasm. |
| 123 | Trim5 | MM5430 | Any process that activates, maintains or increases the rate of autophagy. Autophagy is the process in which cells digest parts of their own cytoplasm. |
| 124 | Mtdh | MM5430 | Any process that activates, maintains or increases the rate of autophagy. Autophagy is the process in which cells digest parts of their own cytoplasm. |
| 125 | Ufl1 | MM5430 | Any process that activates, maintains or increases the rate of autophagy. Autophagy is the process in which cells digest parts of their own cytoplasm. |
| 126 | Gpsm1 | MM5430 | Any process that activates, maintains or increases the rate of autophagy. Autophagy is the process in which cells digest parts of their own cytoplasm. |
| 127 | Ccny | MM5430 | Any process that activates, maintains or increases the rate of autophagy. Autophagy is the process in which cells digest parts of their own cytoplasm. |
| 128 | Atg101 | MM5430 | Any process that activates, maintains or increases the rate of autophagy. Autophagy is the process in which cells digest parts of their own cytoplasm. |
| 129 | Pink1 | MM5430 | Any process that activates, maintains or increases the rate of autophagy. Autophagy is the process in which cells digest parts of their own cytoplasm. |
| 130 | Snx4 | MM5430 | Any process that activates, maintains or increases the rate of autophagy. Autophagy is the process in which cells digest parts of their own cytoplasm. |
| 131 | Dapk1 | MM5430 | Any process that activates, maintains or increases the rate of autophagy. Autophagy is the process in which cells digest parts of their own cytoplasm. |
| 132 | Fbxo7 | MM5430 | Any process that activates, maintains or increases the rate of autophagy. Autophagy is the process in which cells digest parts of their own cytoplasm. |
| 133 | Trim32 | MM5430 | Any process that activates, maintains or increases the rate of autophagy. Autophagy is the process in which cells digest parts of their own cytoplasm. |
| 134 | Optn | MM5430 | Any process that activates, maintains or increases the rate of autophagy. Autophagy is the process in which cells digest parts of their own cytoplasm. |
| 135 | Plekhf1 | MM5430 | Any process that activates, maintains or increases the rate of autophagy. Autophagy is the process in which cells digest parts of their own cytoplasm. |
| 136 | Sting1 | MM5430 | Any process that activates, maintains or increases the rate of autophagy. Autophagy is the process in which cells digest parts of their own cytoplasm. |
| 137 | Larp1 | MM5430 | Any process that activates, maintains or increases the rate of autophagy. Autophagy is the process in which cells digest parts of their own cytoplasm. |
| 138 | C9orf72 | MM5430 | Any process that activates, maintains or increases the rate of autophagy. Autophagy is the process in which cells digest parts of their own cytoplasm. |
| 139 | Atg7 | MM5430 | Any process that activates, maintains or increases the rate of autophagy. Autophagy is the process in which cells digest parts of their own cytoplasm. |
| 140 | Usp20 | MM5430 | Any process that activates, maintains or increases the rate of autophagy. Autophagy is the process in which cells digest parts of their own cytoplasm. |
| 141 | Clec16a | MM5430 | Any process that activates, maintains or increases the rate of autophagy. Autophagy is the process in which cells digest parts of their own cytoplasm. |
| 142 | Svip | MM5430 | Any process that activates, maintains or increases the rate of autophagy. Autophagy is the process in which cells digest parts of their own cytoplasm. |
| 143 | Sesn3 | MM5430 | Any process that activates, maintains or increases the rate of autophagy. Autophagy is the process in which cells digest parts of their own cytoplasm. |
| 144 | Slc35d3 | MM5430 | Any process that activates, maintains or increases the rate of autophagy. Autophagy is the process in which cells digest parts of their own cytoplasm. |
| 145 | Snx7 | MM5430 | Any process that activates, maintains or increases the rate of autophagy. Autophagy is the process in which cells digest parts of their own cytoplasm. |
| 146 | Trim12a | MM5430 | Any process that activates, maintains or increases the rate of autophagy. Autophagy is the process in which cells digest parts of their own cytoplasm. |
| 147 | Calcoco2 | MM5430 | Any process that activates, maintains or increases the rate of autophagy. Autophagy is the process in which cells digest parts of their own cytoplasm. |
| 148 | Atg16l1 | MM5430 | Any process that activates, maintains or increases the rate of autophagy. Autophagy is the process in which cells digest parts of their own cytoplasm. |
| 149 | Hspb8 | MM5430 | Any process that activates, maintains or increases the rate of autophagy. Autophagy is the process in which cells digest parts of their own cytoplasm. |
| 150 | Trim23 | MM5430 | Any process that activates, maintains or increases the rate of autophagy. Autophagy is the process in which cells digest parts of their own cytoplasm. |
| 151 | Kat5 | MM5430 | Any process that activates, maintains or increases the rate of autophagy. Autophagy is the process in which cells digest parts of their own cytoplasm. |
| 152 | Tlr9 | MM5430 | Any process that activates, maintains or increases the rate of autophagy. Autophagy is the process in which cells digest parts of their own cytoplasm. |
| 153 | Trim8 | MM5430 | Any process that activates, maintains or increases the rate of autophagy. Autophagy is the process in which cells digest parts of their own cytoplasm. |
| 154 | Sirt1 | MM5430 | Any process that activates, maintains or increases the rate of autophagy. Autophagy is the process in which cells digest parts of their own cytoplasm. |
| 155 | Cers1 | MM5430 | Any process that activates, maintains or increases the rate of autophagy. Autophagy is the process in which cells digest parts of their own cytoplasm. |
| 156 | Trim6 | MM5430 | Any process that activates, maintains or increases the rate of autophagy. Autophagy is the process in which cells digest parts of their own cytoplasm. |
| 157 | Trim34a | MM5430 | Any process that activates, maintains or increases the rate of autophagy. Autophagy is the process in which cells digest parts of their own cytoplasm. |
| 158 | Deptor | MM5430 | Any process that activates, maintains or increases the rate of autophagy. Autophagy is the process in which cells digest parts of their own cytoplasm. |
| 159 | Sh3bp4 | MM5430 | Any process that activates, maintains or increases the rate of autophagy. Autophagy is the process in which cells digest parts of their own cytoplasm. |
| 160 | Rab3gap2 | MM5430 | Any process that activates, maintains or increases the rate of autophagy. Autophagy is the process in which cells digest parts of their own cytoplasm. |
